# Effects of Atrazine on Chernozem Microbial Communities Evaluated by Traditional Detection and Modern Sequencing Technology

**DOI:** 10.3390/microorganisms9091832

**Published:** 2021-08-29

**Authors:** Fengshan Yang, Mengying Gao, Honggang Lu, Yuning Wei, Huiting Chi, Tai Yang, Mingrui Yuan, Haiyan Fu, Weimin Zeng, Chunguang Liu

**Affiliations:** 1 Engineering Research Center of Agricultural Microbiology Technology, Ministry of Education, College of Life Sciences, Heilongjiang University, Harbin 150800, China; yangfengshan@hlju.edu.cn (F.Y.); fuhaiyan@hlju.edu.cn (H.F.); 2002081@hlju.edu.cn (W.Z.); 2 Heilongjiang Provincial Key Laboratory of Ecological Restoration and Resource Utilization for Cold Region, School of Life Sciences, Heilongjiang University, Harbin 150800, China; 2191227@s.hlju.edu.cn (M.G.); 2141179@s.hlju.edu.cn (H.L.); 2201579@s.hlju.edu.cn (Y.W.); 2201566@s.hlju.edu.cn (H.C.); 2201570@s.hlju.edu.cn (T.Y.); 2211642@s.hlju.edu.cn (M.Y.); 3 Key Laboratory of Microbiology, College of Heilongjiang Province, Harbin 150800, China

**Keywords:** atrazine residue, carbon source utilization efficiency, bacterial population, high-throughput sequencing technology, soil enzyme activity

## Abstract

Atrazine is a long residual herbicide commonly used in maize fields. Although atrazine can effectively control weeds and improve crop yield, long-term application leads to continuous pollution in the agricultural ecological environment, especially in the soil ecosystem, and its impact on soil microorganisms is still not clear. Four methods were used in the experiment to clarify the effect of atrazine on the bacterial populations of cultivated soil layers of chernozem in a cold region in different periods: high-performance liquid chromatography (HPLC), colorimetry, microplate, and high-throughput sequencing. The level of residual atrazine in cold chernozem decreased from 4.645 to 0.077 mg/kg soil over time, and the residue gradually leached into deep soil and then decreased after accumulating to a maximum value. Atrazine significantly affected the activities of urease and polyphenol oxidase activity in the soil layers at different periods but had no significant effect on sucrase and phosphatase activity. Atrazine significantly reduced the diversity of microbial carbon source utilization and total activity in soil layers of 0–10 and 20–30 cm but only reduced the diversity of microbial carbon source utilization in the 10–20 cm layer. Atrazine had no significant effect on bacterial populations (10–12 phyla, 29–34 genera), but had a slight effect on the relative abundance of various groups. Atrazine significantly reduced the diversity of bacterial populations in cultivated soil layers of chernozem in a cold region, and the diversity of bacterial populations decreased with decreased residue. This lays a foundation for guiding the safe use of herbicides on farmland in Northeast China.

## 1. Introduction

Atrazine, a triazine herbicide, has been widely used because of its good herbicidal effect and low cost [1]. However, it can cause soil and water pollution [2] and has a certain influence on the growth and development of sensitive crops and the succeeding crops. Besides, atrazine can interfere with the endocrine system of humans and mammalian animals; it has potential carcinogenic, teratogenic, and mutagenic effects [3]; and it has been listed as a priority pollutant for international environmental control [4]. With regard to protecting human health and ensuring environmental sustainability, the problem of soil and water remediation caused by atrazine pollution has been a significant concern for governments and people all over the world [5]. Chernozem, a type of soil with high fertility, is the most suitable for crop growth in the world [6]. However, due to agricultural intensification over the years, especially the unscientific application of large doses of pesticides on farmland and the evolution of external natural conditions, the content of organic matter in chernozem has decreased [7], the thickness of the soil layer has decreased, and the microecological environment of the soil has been damaged. Soil microorganisms, which act as a sensitive index reflecting soil fertility and quality, have been widely used in ecological safety assessments of pesticide-contaminated soil. At present, there are many studies on soil microbial diversity at home and abroad. However, there is less research on the effect of atrazine on soil microbial diversity, especially in different soil layers. Therefore, we studied this aspect of soil.

Before the 1990s, traditional biochemical methods were mainly used, such as colorimetry, microplates, and microbial culture. In order to evaluate the environmental effect of farmland conversion to secondary forest after atrazine pollution, the soil chemical properties and soil bacterial communities as well as their response to three land use types (primary forest, PF, and SF) were measured by colorimetry. It was found that atrazine residue in SF soil was degraded after natural attenuation for 20 years [8]. The degradation mechanism of sulfadiazine, aureomycin, and atrazine after repeated application in soil samples and the effect of atrazine on the microbial diversity of atrazine-contaminated soil was studied by microplate and MiSeq sequencing methods. The potential internal relationship between atrazine degradation and soil bacterial community structure in atrazine-treated soil was also revealed [9]. The effects of common herbicides on rhizosphere microorganisms in maize fields were studied by the microbial culture method, and the results showed that atrazine had a certain inhibitory effect on bacteria and actinomycetes in the rhizosphere soil [10].

Since the 1990s, with the continuous improvement of scientific and technological methods, there are more and more research technologies on soil microbial diversity, mainly based on modern molecular biological methods such as PCR, DGGE, and high-throughput sequencing technology, which play important roles in the research on atrazine. In one study, cultured soil was collected at different depths in soybean and maize planting areas under different water retention levels. The N_2_O produced by nitrification under the influence of herbicides (chlorsulfuron, atrazine, etc.), the planting system, and protective agriculture was evaluated by gas chromatography and real-time quantitative PCR. The results showed that extensive use of herbicides can promote N_2_O production [11]. The effects of atrazine and lead on soil microbial community, soil net nitrogen mineralization, and atrazine residue were studied by DGGE technology. The results showed that compared with the control group (H = 2.59), the presence of atrazine and lead (especially in high concentrations) to reduced microbial diversity (the lowest H value was 2.23) [12]. High-throughput sequencing of the 16S rRNA gene was used to study bacterial, functional, and community diversity in the rhizosphere. The results showed that although atrazine decreased the richness of the soil bacterial community, the rhizosphere soil had higher bacterial community traits [13].

At present, many methods can be used to study microbial diversity, and each one has advantages and disadvantages. In order to solve the limitations of each method, it is better to use a variety of methods to realize complementary advantages and maximize the advantages and potential of modern molecular biology in soil microbial diversity research, so as to obtain more comprehensive and complete information. The above domestic and foreign studies investigated the influence of atrazine on soil microorganisms from two major aspects, time and space. This research combined the old and new methods, adopted the colorimetric, microplate, and high-throughput sequencing methods, and examined different soil layers and times. The main purpose was to clarify the annual residual level and vertical distribution of atrazine in the soil of the continuous cropping area of Heilongjiang Province in order to reveal the influence of the residual level of atrazine on enzyme activity over time, the bacterial population and abundance, the bacterial function, and the diversity of cultivated soil layers in the maize planting area; to investigate the relationship between atrazine degradation and bacterial population and abundance change; and to contribute to guidance on the safe use of herbicides. We hypothesized the following: (1) applied atrazine will decrease over time and gradually leach into the deep soil, (2) atrazine will affect the diversity and overall activity of microbial carbon source utilization in soil layer, and (3) atrazine may affect the abundance of the soil bacterial population.

## 2. Materials and Methods

### 2.1. Experimental Design and Soil Samples

The soil samples were collected from the continuous cropping experimental maize field of Hulan campus of Heilongjiang University in China, located at 45°59′ N, 126°38′ E. This area is located in the second accumulated temperate zone, with annual accumulated temperature of 2500–2700 °C and annual precipitation of 800 mm. The altitude is 146 m. The design of the plot of the test field was as follows: the minimum area of each cell was 25 m^2^ (5 m × 5 m), surrounded by 2 ridges of protective row, and the interval between communities was 0.5 m. The herbicide was sprayed in an area according to the recommended agricultural quantity (456 g.ai/ha), CK groups were set up without application, and there were three repetitions for each group of tests. The atrazine residues in the previous year were 0.2658 mg/kg soil (0-10 cm), 0.0648 mg/kg soil (10–20 cm), and 0.0252 mg/kg soil (20–30 cm). The soil samples were collected on the first day (d) as well as 7, 21, and 119 d after application of atrazine. After removing dead leaves, stems, and weeds from the topsoil, samples from 0–10, 10–20, and 20–30 cm cultivated soil layers were collected. The five-point sampling method was used to complete the experiment on atrazine in continuous maize cropping. The specific sampling method of the test field is shown in Figure S1.

The physical and chemical properties of the soil were as follows: organic matter 32.2 g/kg, total nitrogen 1.75 g/kg, total phosphorus 0.51 g/kg, total potassium 18.54 g/kg, maximum field water 18.28%, pH 6.31, clay 34.23%, silt 27.29%, and sand 38.48%. After sampling, each soil sample was divided into 2 parts: one was stored in a refrigerator at −80 °C for subsequent sequencing, and the other was dried to remove debris, such as gravel and plant debris, passed through a 0.425–0.250 mm sieve, and stored in a bag in a refrigerator at 4 °C for later use.

### 2.2. Determination of Atrazine Residue in Soil

The soil was pretreated with organic solvent and assessed by liquid chromatography [14]. The HPLC detection conditions were as follows: wavelength: 220 nm; mobile phase: acetonitrile: water = 75:25 (*V*/*V*); flow rate: 0.8 mL/min; column temperature: 30 °C; chromatographic column: Inertsil ODS–3 C18 (4.6 × 250 mm, 5 μm); injection volume: 20 μL. The retention time was 5.4 min.

According to the pretreatment method and instrument conditions, 3 standard solutions of atrazine (1, 2, and 5 mg/kg) were added to the blank soil. The solution was repeated 3 times. Meanwhile, a solution of atrazine acetonitrile was obtained by comparing blank samples. The peak area of atrazine solution was measured by HPLC at 220 nm wavelength. The concentration of atrazine was calculated according to a regression equation. The recovery of atrazine was calculated according to Formula (1):

Addition recovery (%) = (measured concentration/added concentration) × 100%(1)

### 2.3. Determination of Soil Enzyme Activity

In cold chernozem, sucrase activity is measured by 3,5-dinitrosalicylic acid colorimetry [15], urease activity is measured by indophenol blue colorimetry [16], phosphatase activity is measured by sodium diphenyl phosphate colorimetry [15], and polyphenol oxidase activity is measured by pyrogallol colorimetry [15]. Finally, the 4 kinds of soil enzyme activity were detected by an automatic microplate reader.

### 2.4. Functional Diversity of Soil Microbial Community

For the ELISA reaction, the Biolog microplate was removed from the refrigerator in advance and preheated to 25 °C. Then, 10–3 soil diluent was added to the V-shaped tank in the sterile operating room, and an 8-channel pipette gun was used to suck the soil diluent from the tank and inoculate it onto the microplate (150 mL per well µL). Each application was repeated 3 times. The Biolog 96 microplate was self-designed with 3 replicates. A1 = A5 = A9 was the control well without carbon source, and the other 31 wells contained a different carbon source. The classification of the 31 carbon sources is shown in the Supplementary Materials (Table S1). The inoculated microplates were placed in the incubator at constant temperature (25 ± 1 °C), and the culture period was 4, 24, 48, 72, 96, 120, 144, and 168 h. The absorbance values were measured at 590 and 750 nm with the Biolog reader (MicroLog 34.20.05 software) [17]. The total microbial activity index is expressed by the total activity of soil microorganisms and the average well color development (AWCD) of each pore. The AWCD value can reflect the oxidation ability of soil microorganisms and judge their overall carbon source utilization ability. It was calculated according to Formula (2):

(2) $$AWCD=\frac{\Sigma \;\left(A_{i}–A_{A1}\right)}{31}$$

where A_i_ is the absorbance value of hole i (the optical density of each hole), A_A1_ is the absorbance value of hole A1 (the light density value of the control hole), and 31 is the carbon source type of the culture medium. This research type is 31. A_i_ − A_A1_ when the negative value is 0.

The diversity of soil microbial carbon source utilization was analyzed by the Biolog data at 72 h incubation. Shannon, Simpson, and McIntosh diversity indices were used to characterize the functional diversity of soil microbial carbon source utilization. The relative utilization ratio of the soil microbial carbon source is the relative utilization ratio of soil microbes to the 6 carbon sources in the Biolog microplate, which is the percentage of utilization degree of a certain type of carbon source to the 6 carbon sources. The total relative utilization ratio of the 6 carbon sources was 100%, with AWCD as the calculation index.

### 2.5. Determination of Bacterial Population and Abundance in Soil

Genomic DNA was extracted with a kit. According to the designated sequencing region, specific primers with barcodes were designed and synthesized for PCR amplification and product purification. According to the preliminary results of electrophoresis, the concentration of PCR products was determined by a Qubit fluorometric quantitation system. Then, according to the sequencing requirements of each sample, the corresponding proportion of the mixture was homogenized to prepare the MiSeq library, and then MiSeq high-throughput sequencing was performed [10].

### 2.6. Statistical Analysis

Microsoft Excel 2020 software was used to carry out basic statistical processing, such as average value, standard deviation of experimental data, and draw pictures; SPSS 16.0 software was used for one-way ANOVA; and the Duncan method was used to test the differences in AWCD value, soil microbial diversity index, and carbon source utilization rate.

## 3. Results

### 3.1. Residue Dynamics of Atrazine in Different Plough Layers of Chernozem in Cold Region

#### 3.1.1. Determination of Standard Addition Recovery Test

According to the results of HPLC, the standard curve of atrazine, y = 107,653x + 5206.5, R² = 0.9999, was successfully established. The results show that the recovery of atrazine ranged from 90.17 to 104.69%, with RSD between 0.79 and 2.90%, which met the requirements of pesticide residue analysis.

#### 3.1.2. Residue Changes of Atrazine in Different Plough Layers of Chernozem in Cold Region

**Figure 1:**
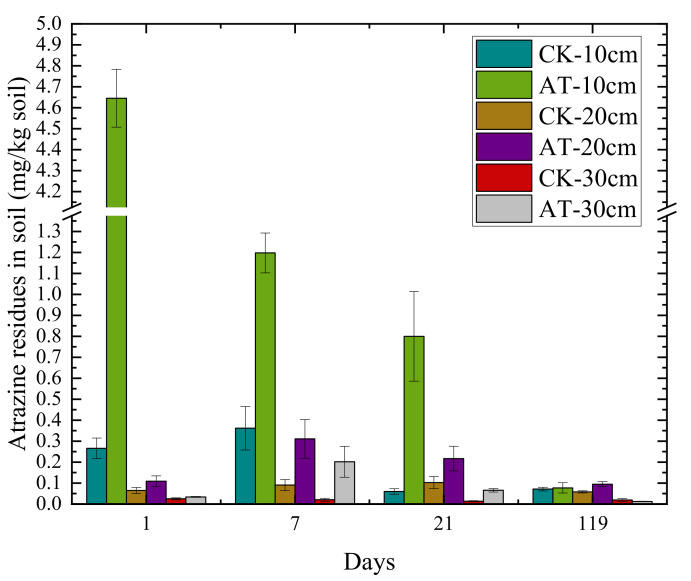
Changes in atrazine residue in various soil layers at different periods after application. Each value represents mean ± SD (*n* = 3).

The total atrazine residue in the 0–10 cm soil layer of cold chernozem decreased with increased application time and gradually leached into the deep soil. The atrazine residue in the 10–20 and 20–30 cm soil layers gradually accumulated, reaching the maximum at 7 d after application, and then decreased gradually. The residual digestion curve equation for atrazine in the pesticide residue test in the 3 soil layers in chernozem, 0–10, 10–20, and 20–30 cm, was y = 2.2423 × e^−0.029x^, R² = 0.8899; y = 0.3556 × e^−0.011x^, R² = 0.8919; and y = 0.1606 × e^−0.022x^, R² = 0.9129, respectively. The half-life of atrazine in the 3 soil layers was 30.91, 194.88, and 431.51 d, respectively. In the 0–10 cm soil, the rate of atrazine digestion was 98.34% at 119 d. At 10–20 and 20–30 cm, the digestion rate was negative at 7 d, indicating that the atrazine residue increased, reached its maximum at 7 d, and then decreased gradually. At 119 d, the rate of atrazine degradation in the 20–30 cm soil layer reached 93.68%, while that in the 10–20 cm soil layer reached only 76.86%, indicating that atrazine would remain in the soil for a long time and cause harm to subsequent crops. The amount of atrazine residue in the soil layers, from high to low, was 0–10 cm > 10–20 cm > 20–30 cm (Figure 1).

### 3.2. Effect of Atrazine on Enzyme Activity in Chernozem

**Figure 2:**
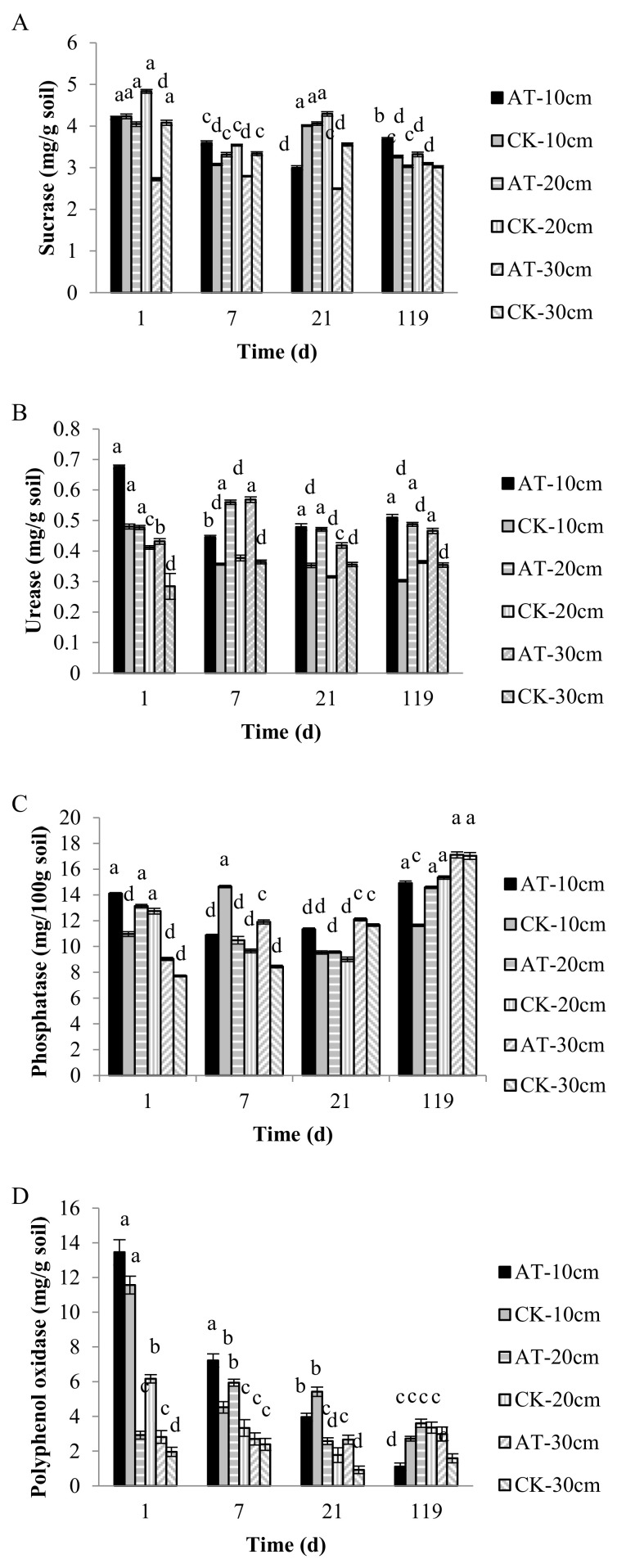
Effect of atrazine on enzyme activity in cultivated soil layers in different periods in chernozem of cold region. (**A**) Sucrase, (**B**) urease, (**C**) phosphatase, (**D**) polyphenol oxidase.Each value represents mean ± SD (*n* = 3). There is no significant difference between CK and AT groups (*p* > 0.05), but there is a significant difference between CK and AT groups (*p* < 0.05). Where a, b, c, d means that there is no significant difference with the same letter, and there is significant difference with different letters.

The concentration of sucrase in the 0–10 cm soil layer in AT groups was 4.198 mg/g and in CK groups was 4.234 mg/g on the first day of application. At 7 d, the concentration of sucrase was 3.079 mg/g in AT groups and 3.596 mg/g in CK groups; at 21 d, the concentration was 2.998 mg/g in AT groups and 4.011 mg/g in CK groups; and at 119 d, the concentration was 3.680 mg/g in AT groups and 3.270 mg/g in CK groups. From the above data, it can be seen that in the 0–10 cm soil layer, the change in sucrase activity of AT groups was the same as that of CK groups, which shows that atrazine had little effect on the sucrase activity in soil. In terms of the overall sucrase activity in the three soil layers, it can be seen that in the CK groups, the activity in the 10–20 cm soil layer was significantly higher than in the other two soil layers (Figure 2A). The urease activity of AT groups with atrazine application was significantly higher than that of CK groups, and the change trend of urease activity with atrazine in the 0–10, 10–20, and 20–30 cm soil layers in cold chernozem is consistent with the change trend of atrazine residue in the three soil layers in the same period, which indicates that atrazine stimulates increased urease activity in soil (Figure 2B).

The results for phosphatase show that the concentration of phosphatase in AT groups was 14.098 mg/100g and in CK groups was 10.968 mg/100g in the 0–10 cm soil on the first day. At 7 d, the phosphatase concentration was 10.880 mg/100g in AT groups and 14.65 mg/100g in CK groups; at 21 d, the concentration was 11.33 mg/100g in AT groups and 9.54 mg/100g in CK groups; and at 119 d, the concentration was 14.910 mg/100g in AT groups and 11.64 mg/100g in CK groups. AT and CK groups showed upward and downward fluctuation in the three soil layers. The data show that atrazine had no effect on phosphatase activity in the different soil layers of chernozem. With regard to total phosphatase activity, it can be seen that there is no obvious change rule in the three soil layers, which is almost in a natural fluctuation state (Figure 2C).

The results for polyphenol oxidase in cold chernozem show that the change trend of activity in two soil layers (0–10 and 10–20 cm) is consistent with that of atrazine in the same soil layer at the same time, indicating that atrazine stimulates increased polyphenol oxidase activity in soil. The polyphenol oxidase activity in AT groups at 20–30 cm was significantly higher than in CK groups, indicating that atrazine could stimulate increased polyphenol oxidase in soil and improve soil fertility (Figure 2D). In a word, atrazine can stimulate polyphenol oxidase in different soil layers at different periods in cold chernozem. Over time, the total polyphenol oxidase activity in soil gradually decreases.

### 3.3. Effect of Atrazine on Carbon Utilization of Microbial Community in Chernozem in Cold Region

#### 3.3.1. Average Color Change Rate (AWCD) of Soil Microorganisms in Plough Layers in Different Periods

In the 0–10 cm soil layer, the AWCD of CK groups was significantly higher than that of AT groups after 1 d application, which may be due to the inhibition of soil microbial activity by a high concentration of atrazine; both groups were the same in the 20–30 cm soil layer as the 0–10 cm layer, and showed the highest microbial activity; at 20–30 cm, AT groups were higher than CK groups, which may be due to the leaching of atrazine into this layer. Low-dose atrazine promotes increased soil microbial activity, and the total activity of the three soil layers was in the order of 10–20 cm > 0–10 cm > 20–30 cm. At 7 d, CK groups were significantly higher than AT groups in 0–10 cm, but the difference between the two groups was smaller than on the first day. It may be that the application of high-concentration atrazine inhibited the activity of soil microorganisms, but over time, part of the atrazine leached to the next soil layer, so the inhibition effect was reduced.

In the 10–20 cm soil layer, AT groups were slightly higher than CK groups, which may be due to the continuous leaching of atrazine in the upper layer, resulting in increased atrazine concentration in this layer, which slightly inhibited the soil microbial activity. In the 20–30 cm soil layer, CK groups were significantly higher than AT groups, which may be due to the increased drug concentration caused by atrazine leaching, inhibiting the soil microbial activity in this layer. In this period, the total soil microbial activity from high to low was 0–10 cm > 10–20 cm > 20–30 cm.

**Figure 3:**
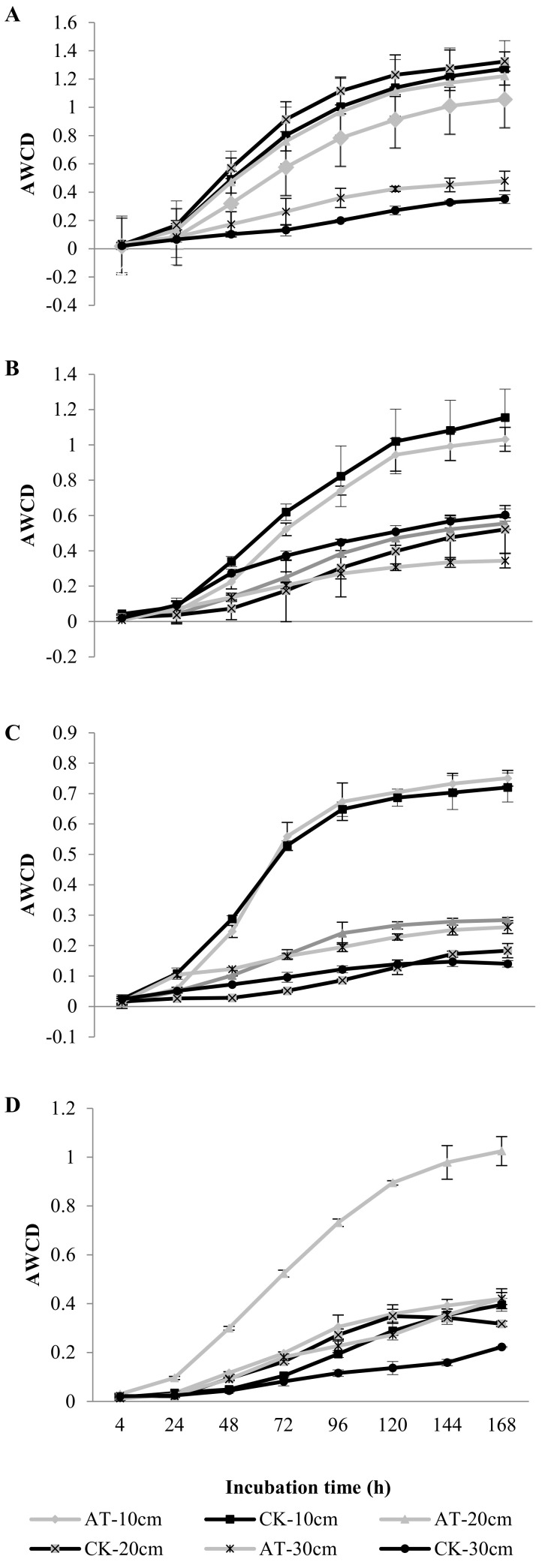
Effect of atrazine on AWCD of microbial community in cultivated soil layers of chernozem. Each value represents mean ± SD (*n* = 3). (**A**) 1 d, (**B**) 7 d, (**C**) 21 d, and (**D**) 119 d.

After 21 d of application, with decreased atrazine concentration in the three soil layers, AT groups slightly exceeded CK groups, probably because the atrazine had gradually leached to the deeper soil layer, and soil microbial activity had recovered and even increased under the lower concentration of atrazine. The total activity in the three soil layers from high to low was 0–10 cm > 10–20 cm > 20–30 cm. After 119 d, the soil biodiversity of AT and CK groups in the 0–10 and 20–30 cm soil layers was almost the same, indicating that the effect of atrazine application had been eliminated in those two layers. In the 10–20 cm soil layer, AT groups were significantly higher than CK groups, probably because atrazine residue in this layer was higher than in the other two layers, but it was related to low concentration, which stimulated the microbial activity in this layer. In general, the soil microbial activity in the three soil layers from high to low was 10–20 cm > 0–10 cm > 20–30 cm (Figure 3). The effect of atrazine on the microbial community index of different tillage soil layers in chernozem is shown in Figure S2. The effect of soil microbial community on relative utilization efficiency of different carbon sources is shown in Figure S3.

#### 3.3.2. Correlation between Soil Microbial Community, Enzyme Activity, Carbon Source Utilization, and Atrazine Residue

According to the correlation analysis of atrazine residue, soil enzyme activity, and microbial carbon source utilization functional diversity (Table 1), the results show that atrazine was significantly related to urease and polyphenol oxidase at the 0.01 level, and there was also a significant correlation between urease and polyphenol oxidase, which indicates that atrazine could increase their activity. Polyphenol oxidase was significantly related to the carbon source of carboxylic acid by AWCD at the 0.01 level, and sucrase enzyme was significantly correlated with AWCD, McIntosh index, atrazine and carboxylic acid carbon source, amino acids, and McIntosh index at the 0.05 level. There were significant correlations among the Shannon, Simpson, and McIntosh indices. There was a significant negative correlation between atrazine and the Shannon and Simpson indices (*p* < 0.01), which indicates that atrazine could inhibit the diversity of microorganisms. The Shannon index had a significant negative correlation with carboxylic acid carbon sources (*p* < 0.01), urease (*p* < 0.01), and sucrase enzyme (*p* < 0.05).

### 3.4. Effects of Atrazine on Bacterial Diversity in Cultivated Soil Layers

#### 3.4.1. Effects of Atrazine on Bacterial Composition in Tillage Soil Layers of Chernozem in Cold Region

The effect of atrazine on the bacterial population in different plough layers of cold chernozem was analyzed. The composition of the microbial community of atrazine in different periods and plough layers of cold chernozem was basically the same. In the 0–10 cm soil layer, there were 12 main phyla of bacteria (Figure S4A) and 29 genera, including *Kaistobacter*, *Nitrospira*, DA101, *Flavisolibacter*, *Candidatus Solibacter*, and *Rhodoplanes*, and the relative abundance of these six genera was about 70%, which makes them the dominant microbiota in this soil layer. In the 0–10 cm soil layer, eight genera (*Acinetobacter*, *Aeromicrobium*, *Streptomyces*, *Stenotrophomonas*, *Delftia*, *Nocardioides*, *Candidatus Koribacter*, *Kribbella*) in the AT groups were consistent with the residual change of atrazine in the soil. On the first day of application, the relative abundance was higher than that of the CK groups in the same period, showing an increasing trend, and at 7 d of application, the relative abundance increased to the maximum (higher than that of the CK groups in the same period). The relative abundance of atrazine reached the minimum or remained stable at 119 d. AT groups showed the same trend as CK groups in four other periods after application, but the increase and decrease of AT groups were more significant than CK groups.

**Figure 4:**
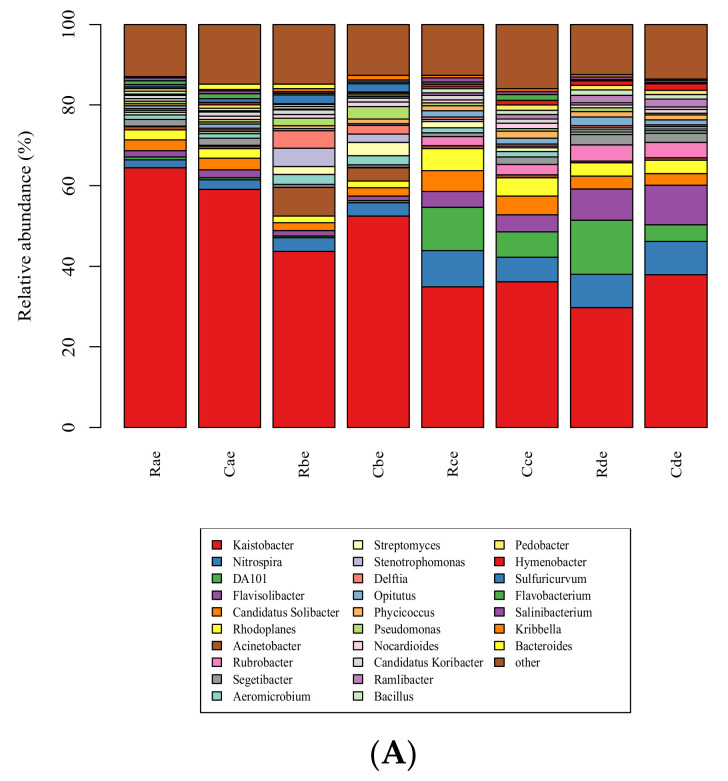

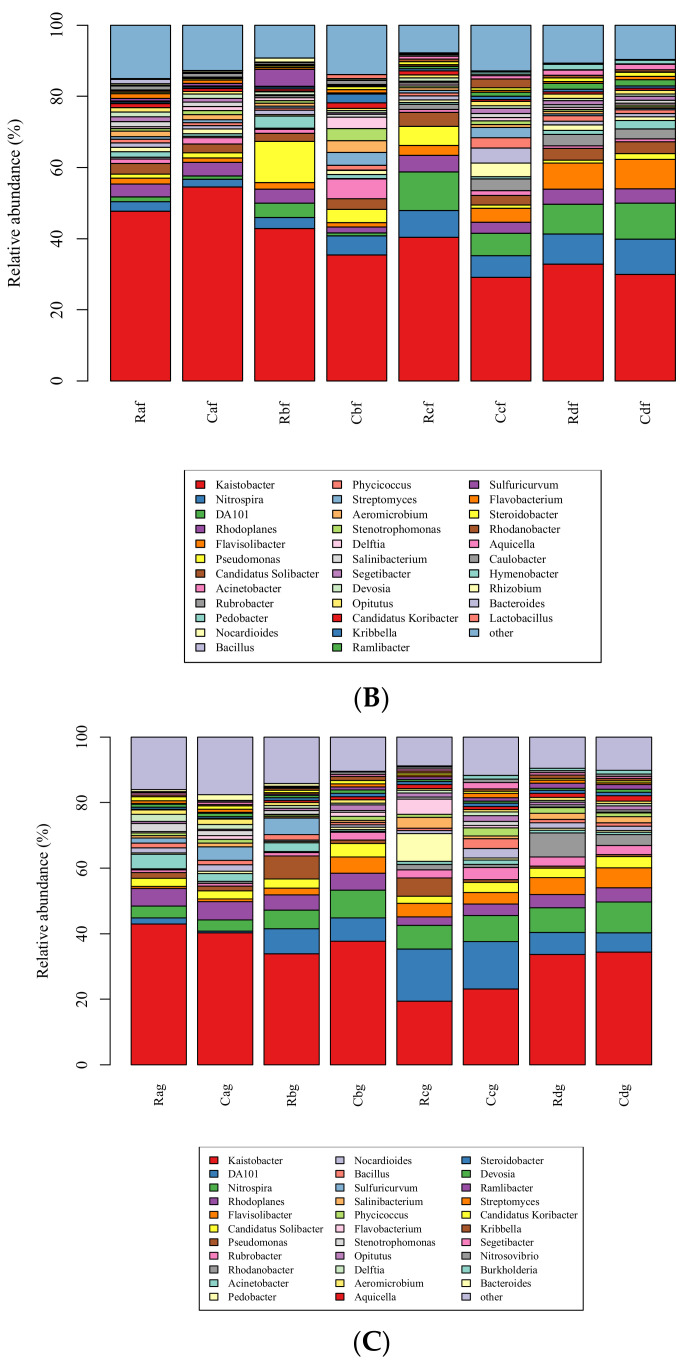
Community populations of samples in soil layers at the genus level. (**A**) 0–10 cm, (**B**) 10–20 cm, (**C**) 20–30 cm. Each value represents mean ± SD (*n* = 3). R, C indicate atrazine at recommended dose in AT and CK, respectively; a, b, c, d indicate soil samples at 1, 7, 21, and 119 d, respectively; e, f, g indicate 0–10, 10–20, and 20–30 cm soil levels, respectively.

On the contrary, in the 0–10 cm soil layer, seven genera of bacteria (DA101, *Flavisolibacter*, *Rubrobacter*, *Opitutus*, *Hymenoptera*, *Ramlibacter*, *Bacillus*) in AT groups were not consistent with the residual change of atrazine in the soil. The relative abundance of seven genera of bacteria was lower than in the CK groups on the first day and decreased to the lowest at 7 d. After that, the residual amount of atrazine in the soil layer decreased continuously until 21 d and 119 d, and the relative abundance of seven genera of bacteria reached the maximum or remained stable at 119 d. The AT groups showed the same trend as the CK groups in four other periods after application, but the increase and decrease of AT groups was more significant. In addition, the relative abundance of *Kaistobacter* and *Flavobacterium* decreased by 78.6 and 85.6% in the two groups, respectively, over time, while *Nitrospira* increased by 91.9% at 21 d and increased to the maximum value, while the relative abundance of *Phycoccus* remained unchanged (Figure 4A).

In the 10–20 cm soil layer, there were 10 phyla (Figure S4B) and 34 genera of bacteria, and the relative abundance of seven genera (*Kaistobacter*, *Nitrospira*, *DA101*, *Rhodoplanes*, *Flavisolibacter*, *Pseudomonas*, *Candidatus Solibacter*) accounts for about 60%, which makes them the dominant microbiota in this soil. In this soil layer, the relative abundance of five genera of bacteria (*Rhodoplanes*, *Pseudomonas*, *Stenotrophomonas*, *Delftia*, *Kribbella*) in the AT groups was consistent with the residual change of atrazine in the soil. The change trend of *Rubrobacter*, *Nocardioides*, and *Ramlibacter* was the opposite. In addition, the relative abundance of nine genera of bacteria (*Kaistobacter*, *Acinetobacter*, *Bacillus*, *Streptomyces*, *Aeromicrobium*, *Salinibacterium*, *Devosia*, *Flavobacterium*, *Caulobacter*) in the AT groups showed a continuous downward trend over time, while *Flavisolibacter* and *Aquicella* continued to rise (Figure 4B).

In the 20–30 cm soil layer, there were 10 phyla (Figure S4C) and 32 genera of bacteria. Among them, the relative abundance of eight genera (*Kaistobacte*, DA101, *Nitrospira*, *Rhodoplanes*, *Flavisolibacter*, *Candidatus Solibacter*, *Pseudomonas*, *Rubrobacter*) was more than 60%, which makes them the dominant microbiota in this soil layer. In this soil layer, the relative abundance of seven genera (*Candidatus Solibacter*, *Acinetobacter*, *Bacillus*, *Delftia*, *Devosia*, *Stenotrophomonas,* and *Candidatus Koribacter*) in the AT groups decreased continuously over time, and *Rhodanobacter*, *Ramlibacter*, and *Burkholderia* increased continuously. In addition, the variation trend of relative abundance of two genera (*Pseudomonas* and *Steroidobacter*) in the AT groups was consistent with that of atrazine residue in soil.

The trend of *Nocardiosis*, *Phycicococcus*, and *Streptomyces* was the opposite. However, at the genus level, the relative abundance of *Kaistobacter* in the 0–10 and 10–20 cm soil layers decreased continuously with a decrease in atrazine residue at 1, 7, 21, and 119 d; its relative abundance also decreased with decrease in atrazine residue in the 20–30 cm soil layer, and the minimum value appeared at 21 d. In addition, the relative abundance of *Kaistobacter* decreased with increased soil layers at 1, 7, 2, and 119 d. The relative abundance was higher in CK groups than AT groups (Figure 4C).

The influence of atrazine on the heatmap clustering of genera in cultivated layers of chernozem in a cold region was analyzed as follows. According to the genera composition and relative abundance of each sample in the chernozem in different periods and layers, genera heatmap analysis was carried out, and the 30 genera with the highest abundance were extracted for heatmap clustering analysis at the taxonomic level. In the cluster diagrams, each small square represents each genus, and its color represents the abundance of the genus. The greater the abundance, the darker the color (red is large and green is small). Each row represents the abundance of each genus in different samples, and each column represents the abundance of all genera in each sample. The upper tree shows the clustering analysis results of different samples from different experimental groups, and the left tree shows the clustering analysis results of different genes from different samples. In different soil layers, with the application of atrazine, the effect on the bacterial abundance of CK and AT groups at the genus level showed a change trend from insignificant to significant and then returned to the CK level. The left is divided into six clusters and the top is divided into two clusters.

**Figure 5:**
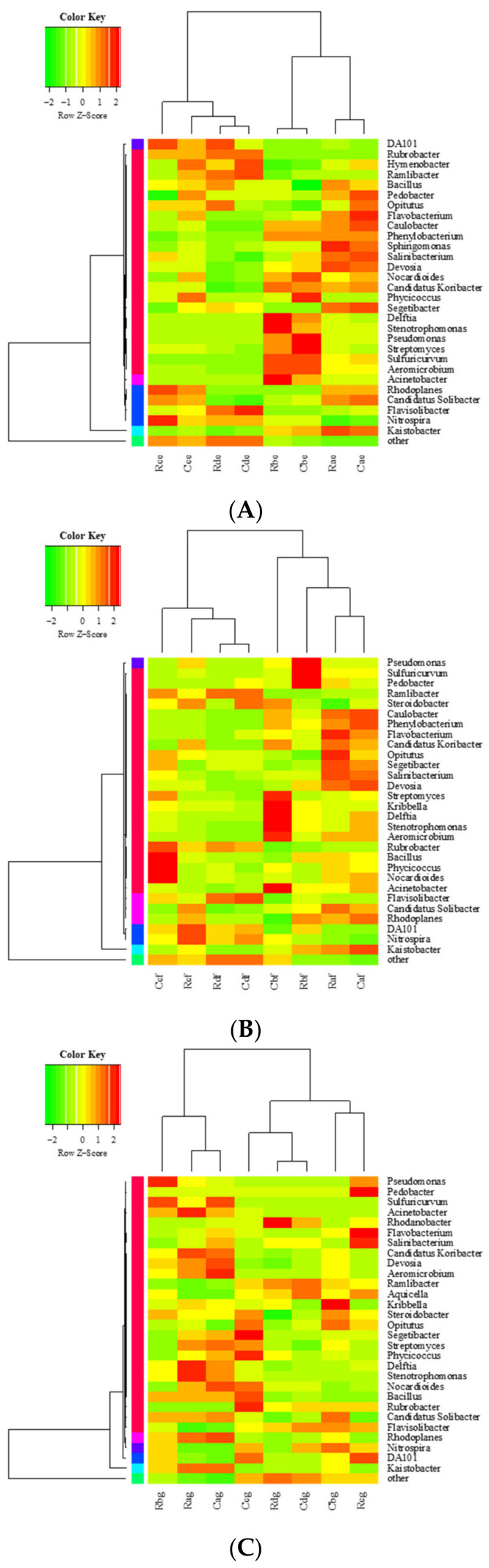
Heatmap of top 30 microbial genera in soil layers. (**A**) 0–10 cm, (**B**) 10–20 cm, (**C**) 20–30 cm. Each value represents mean ± SD (*n* = 3). R, C indicate atrazine at recommended dose in AT and CK, respectively; a, b, c, d indicate soil samples at 1, 7, 21, and 119 d, respectively; e, f, g indicate 0–10, 10–20, and 20–30 cm soil levels, respectively.

At 0–10 cm, different samples are divided into two clusters, of which 1 and 7 d are a cluster and 21 and 119 d are a cluster. The application of atrazine had no significant effect on the bacterial abundance of CK and AT groups at the genus level at 1, 7, and 119 d and had a significant effect on the bacterial abundance at 21 d. At 10–20 cm, different samples are divided into two clusters, of which 1 and 7 d are a cluster and 21 and 119 d are a cluster. The application of atrazine had no significant effect on the bacterial abundance of CK and AT groups at the genus level at 1 and 119 d but had a significant effect on the bacterial abundance at 7 and 21 d. At 20–30 cm, different samples are divided into two clusters, of which 1 and 7 (AT) d are a cluster and 7 (CK), 21, and 119 d are a cluster. The application of atrazine had no significant effect on the bacterial abundance of CK and AT groups at the genus level at 1 and 119 d but had a significant effect on the bacterial abundance at 7 and 21 d (Figure 5 and Figure S5).

#### 3.4.2. Effect of Atrazine on Diversity of Single Samples in Different Plough Layers of Chernozem in Cold Region

The effect of atrazine on bacterial alpha diversity in different plough layers of cold chernozem was analyzed as follows. Alpha diversity analyzes the species diversity in a single sample. Based on the results of OTU, Shannon index, Chao1 index, phylogenetic diversity (PD whole tree), and observations, the number of species was calculated for biodiversity analysis. The Chao and ACE indices do not consider species abundance, only species number. The Shannon index represents species abundance and evenness. The larger the observed number of species and the PD whole tree index, the richer the species. The larger the values of the above five indexes, the higher the species diversity of the samples.

**Figure 6:**
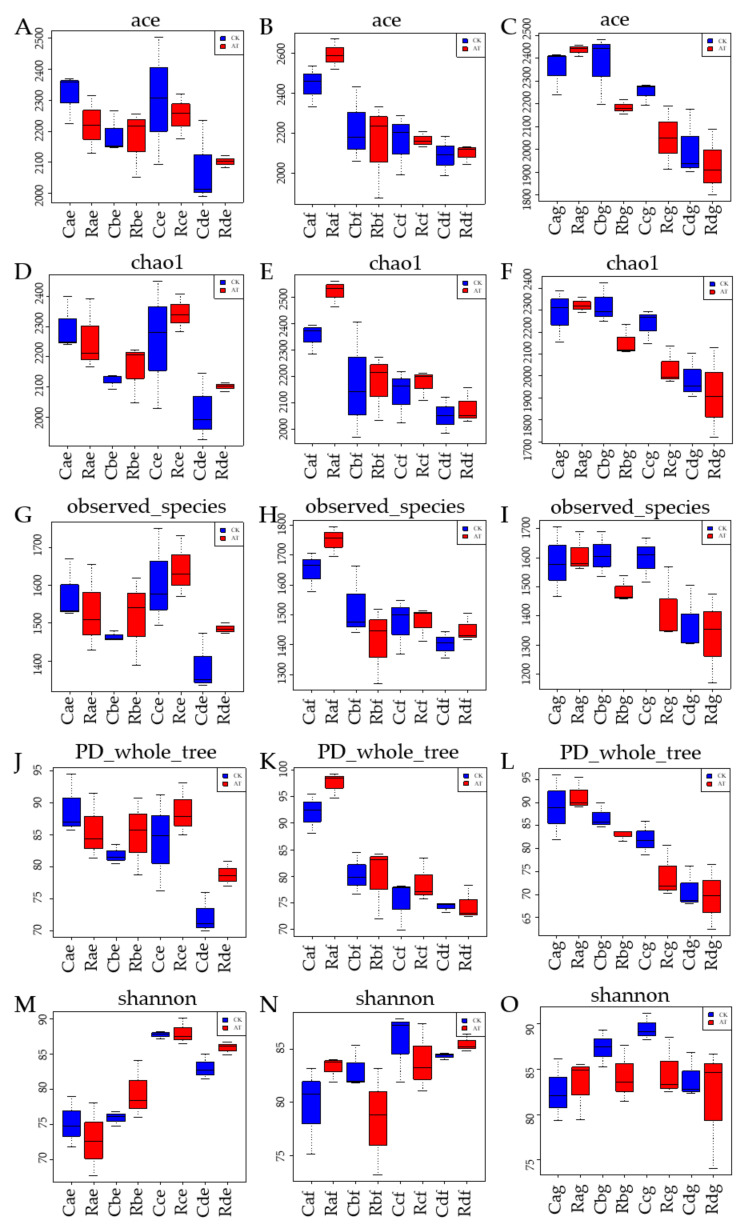
Variation of alpha diversity in different soil layers of atrazine-contaminated soil. (**A**,**D**,**G**,**J**,**M**) 0–10 cm; (**B**,**E**,**H**,**K**,**N**) 10–20 cm; (**C**,**F**,**I**,**L**,**O**) 20–30 cm. Each value represents mean ± SD (*n* = 3). R, C indicate atrazine in AT and CK, respectively, at recommended dose; a, b, c, d indicate soil samples at 1, 7, 21, and 119 d, respectively; e, f, g indicate 0–10, 10–20, and 20–30 cm soil level, respectively.

The alpha diversity differences (ACE, Chao1, observed number of species, PD whole tree, Shannon, etc.) of atrazine pollution in the 0–10, 10–20, and 20–30 cm soil layers were determined. The ACE value in the 0–10 cm layer was lower than the CK groups on the first day of application and was restored and higher than the CK groups at 7 d. At 21 d, ACE reached the maximum value (highest peak), then gradually decreased to the lowest value but was still higher than the CK groups at 119 d. The observed number of species showed the same trend, but the change was more significant, and the difference was greater compared to the ACE value. The same trend was observed for the number of species in the Chao1 index, but the difference was not significant. The Shannon index had the same trend, and the difference was insignificant compared with the observed number of species and Chao1 index. Moreover, it was not significant compared with CK groups. The PD whole tree index showed the same trend, and the difference was significant. In conclusion, the five alpha diversity indices showed the same trend for diversity of microorganisms in soil in the 0–10 cm layer polluted by atrazine: inhibition–weakened inhibition–gradual recovery–increase at 1, 7, 21, and 119 d (Figure 6A,D,G,J,M).

In the 10–20 cm soil layer, the alpha diversity index showed that the ACE value was higher than that of the CK groups at 1 d, and decreased gradually at 7 d, but was still higher. At 21 d of application, the ACE value decreased to the lowest value and then gradually returned to the control level or even higher. The Chao1 index showed the same trend, but the difference was not significant. The PD whole tree index showed the same trend as ACE, Chao1, and observed number of species, and the difference was significant. The Shannon index was higher for AT groups than CK groups on the first day of application and decreased to the lowest value rapidly on the seventh day. Then, it gradually recovered and then quickly recovered to the first-day level after 21 d, but was still lower compared to CK groups in the same period. It then gradually recovered to the control level and became higher than the level of CK groups at 119 d, and the difference was very significant. In conclusion, the five alpha diversity indices had the same microbial carbon source utilization diversity in atrazine-contaminated soil in the 10–20 cm layer; i.e., inhibition–enhanced inhibition–inhibition–increase at 1, 7, 21, and 119 d, respectively (Figure 6B,E,H,K,N).

In the 20–30 cm soil layer, the alpha diversity index showed that the ACE value was higher compared to the CK groups on the first day and decreased continuously, and it was lower compared to the CK groups for the first time at 7 d. After 21 and 119 d of application, the ACE value continued to decrease and was lower compared to the CK groups. The observed number of species, PD whole tree, and Shannon index were significantly different from ACE and Chao1 from 1 to 21 d, but their diversity index was higher than that of CK groups at 119 d, indicating that the diversity of the three indices had recovered. In conclusion, the five alpha diversity indices had basically the same variation trend of microbial carbon source utilization diversity in atrazine-contaminated soil in the 20–30 cm layer; i.e., increase–inhibition–decrease–increase at 1, 7, 21, and 119 d (Figure 6C,F,I,L,O).

#### 3.4.3. Effects of Atrazine on Diversity of Multiple Samples in Different Plough Layers of Chernozem in Cold Region

Beta diversity was used to analyze species diversity differences among samples. As a measure of beta diversity, UniFrac was used to compare species community differences with phylogenetic distance information.

**Figure 7:**
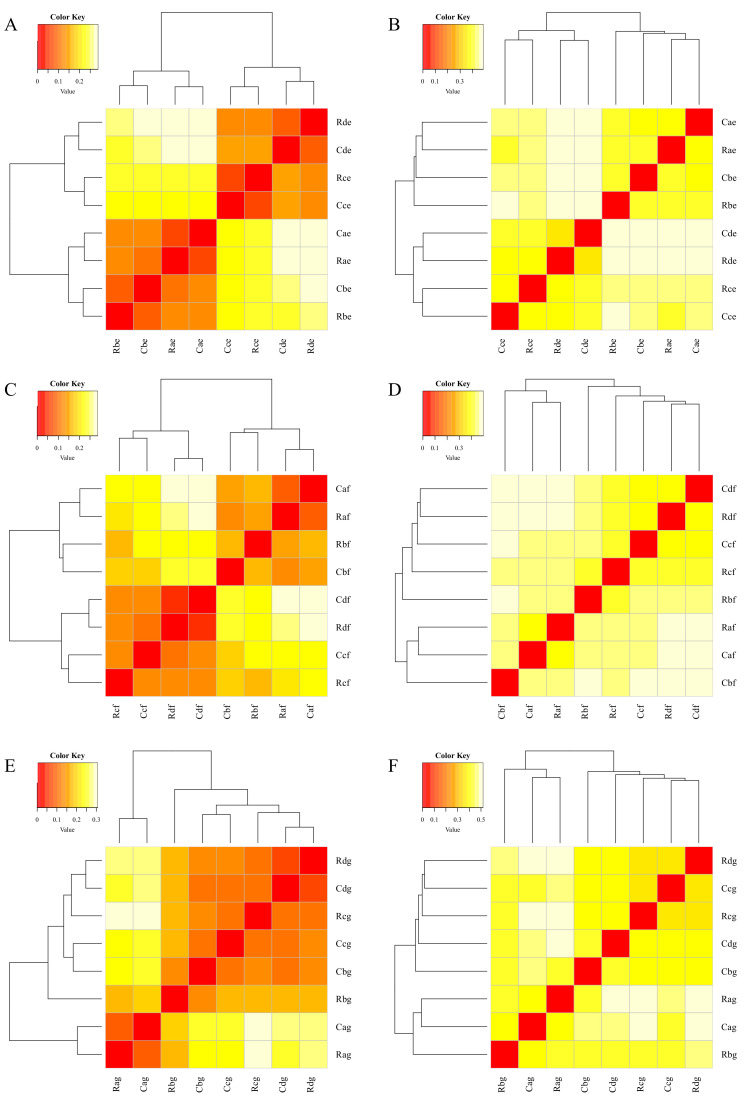
Soil layer abundance thermograms. (**A**,**C**,**E**) Weighted_unifrac; (**B**,**D**,**F**) unweighted_unifrac. (**A**,**B**) 0–10 cm; (**C**,**D**) 10–20 cm; (**E**,**F**) 20–30 cm. Each value represents mean ± SD (*n* = 3). R, C indicate atrazine and control group, respectively, with recommended dose; a, b, c, d indicate soil samples at 1, 7, 21, and 119 d after spraying, respectively; e, f, g indicate soil samples at 0–10, 10–20, and 20–30 cm soil level, respectively.

The beta diversity of samples in different cultivated soil layers of chernozem in a cold region was analyzed according to weighted and unweighted UniFrac. The results show that in soil layers of 0–10, 10–20, and 20–30 cm, with increased atrazine application time, the evolutionary distance between species gradually increased, indicating that the differences among samples became larger. However, with decreased atrazine residue in the later stage, the species distance between samples gradually decreased and the difference became smaller. The variation trend of CK and AT groups was the same, and there was no significant difference in the evolutionary distance between them, indicating that atrazine had no significant effect on the beta diversity of species community in different cultivated soil layers (Figure 7). Principal component analysis of samples from the 3 soil layers is shown in Figure S6. An intergroup difference analysis of atrazine in the samples is shown in Figure S7.

## 4. Discussion

### 4.1. Dynamic Residue of Atrazine in Different Plough Layers of Chernozem in Cold Region

The study of atrazine residue can help us to understand the effect of atrazine on soil microorganisms and then to apply it scientifically. In this experiment, the residual amount of atrazine in cultivated soil layers in the continuous maize cropping area of Heilongjiang Province was determined by HPLC. It can be seen from the quantity that the residual level of atrazine in the chernozem of this cold region gradually reduces over time. From the vertical distribution of soil, the residue will gradually leach to the deep soil, where it accumulates, and when the accumulation reaches the maximum value, it will gradually decrease. When pesticide is applied to soil, there is an adsorption effect, which causes pesticide residue to remain in the soil. Because of the weak adsorption of atrazine in soil, it has strong mobility, and factors such as rainfall, plant root adsorption, and microbial activity in the external environment lead to its leaching into deep soil.

The decline of atrazine in soil is affected by many factors in the environment, among which microbial degradation is important for pesticide degradation in soil [18]. The digestion law of atrazine in soil layers in different periods basically conforms to the first-order dynamic law. The digestion rate of atrazine in soil layers of 0–10 and 20–30 cm in chernozem reached 93%, with a half-life of 30.19 and 431.51 d, respectively. This result is basically consistent with the degradation dynamics of atrazine in Shenyang and Xinmin soil (anthrosols) [19] and is significantly different from the half-life of atrazine in Jiaozuo and Jinan soil (cambisols) [20]. In another study, atrazine was applied to natural grassland and farmland floodplain soil in the Liverpool plain (podzols), New South Wales, in order to observe its degradation rate and yield related to soil properties, including microbial community analysis by t-RFLP. The soils differed in their atrazine treatment history. The degradation rate (half-life) in cropped soil was more rapid (2 to 7 days) than in two grassland soils (8 to 22 days) [21].

The degradation rate in this study may have been caused by the different sources of soil and the climate conditions of the environment. The degradation rate of atrazine in the 10–20 cm soil layer was only 76.86%, and the half-life was 194.88 d. This shows that atrazine does not degrade easily, mainly relying on the biodegradation of alkylation, hydrolysis, and ring opening. The degradation of atrazine in soil will be directly affected by the decomposition and content of soil organic matter and the retention of residues [22]. At the same time, it is also affected by comprehensive factors such as the initial concentration of atrazine, soil microorganisms, soil type, pH, soil temperature, and humidity. In addition, Heilongjiang Province has long, cold winters, the microbial activity is low in frozen soil, and the sunshine time is short in autumn and winter, when atrazine does not volatilize. Photolysis occurs only on the soil surface. The photolysis speed in acid and alkaline soil is faster than that in neutral soil. Because of the weak UV intensity and shallow photodegradation depth in the field, photolysis is slow, which leads to the prolonged time in the soil layer [23].

### 4.2. Effect of Atrazine on Enzyme Activity in Cultivated Soil Layers of Chernozem in Cold Region

Soil enzymes are very important biocatalysts in the process of soil metabolism. A pot experiment with luvisols scraped from the 0–15 cm soil layer of a peanut crop field in rural Andhra Pradesh, India, showed that atrazine application could reduce the soil enzyme activity [24]. In the experiment in our study, four enzymes were analyzed: sucrase, urease, phosphatase, and polyphenol oxidase. In determining sucrase activity in cold chernozem, it is related to many soil factors such as the levels of nitrogen, phosphorus, and organic matter, the number of microorganisms, and the soil respiration intensity, which can reflect the intensity of microorganism activity as well as soil maturity and fertility. In this study, sucrase was not affected by atrazine and its residues in soil. This may be due to the fact that sucrase in soil is an extracellular enzyme that comes from plant roots or microorganisms but is not affected by the growth and reproduction of soil microorganisms.

Atrazine has a certain stimulating effect on urease, which may be why it can be used as a nitrogen source for microbial growth, thus improving urease activity. Soil samples from the Kogi State University farm (ferralsols) were treated with herbicides at rates recommended by the manufacturer in concentrations above and below the recommended rates. As the concentration of herbicides increased, the urease activities increased correspondingly, with butachlor-treated soils having more urease activities than atrazine-treated soils [25]. This result is contrary to the result showing that atrazine inhibits the urease activity of chernozem among the three herbicides [26]. The reason may be that there were differences in the tested soil, and some differences between the field test and the laboratory simulation test. This result is also different regarding the effect of atrazine on soil urease under two fertilization conditions [27]. It was shown that high concentrations of atrazine can significantly inhibit urease activity, and the inhibition degree is positively correlated with the concentration; however, low concentrations stimulated urease to a certain extent, and then inhibited it. This may be related to the differences in soil samples, application methods, and environmental conditions.

The effect of atrazine on soil urease activity by the indoor culture method was studied [28]. The results showed that during the culture, atrazine had an active inhibition–activation effect on soil urease. The reason for this change may be that in the early stage of culture, the main components of soil have a certain buffer capacity for atrazine pollution. The adsorption of the solid phase composition lowers the content of atrazine in soil, and the effect on urease is activated. In the middle of the culture, atrazine and its degradation products contain -NHR and other functional groups. These functional groups can interact with organic matter, partially covering up and occupying the activity center of urease and preventing the combination of urea and the enzyme activity center, which leads to a decreased enzyme reaction rate and competitive inhibition with the substrate [29]. Soil urease activity shows an inhibition effect; atrazine is fixed, decomposed, or loses its biological toxicity effect on soil microorganisms. The microorganisms gradually produce resistance to atrazine, and the urease activity gradually returns to normal [30]. The results of the indoor culture method showed that atrazine had different activation–inhibition–activation effects on soil phosphatase during the culture period [31].

Phosphatase is an important index to evaluate soil phosphorus biotransformation and is very important in accelerating the dephosphorization rate and improving the effectiveness of soil phosphorus. Unlike the Kogi State University farm test, another test showed that phosphatase activities decreased with increased herbicide concentration [25]. This may be related to differences in culture time, soil samples, field test, indoor application method, and external environmental conditions. In addition, atrazine also has a certain stimulation effect on the polyphenol oxidase activity in soil, which is consistent with the results obtained in a study on the defensive enzyme reaction of resistant soybean varieties to atrazine [32]. Many factors affect soil enzyme activity, such as the physical and chemical properties of soil, soil microorganisms, soil nutrients, agricultural vegetation, fertilization and other agricultural measures, plant roots, and human factors. When the physical properties, temperature, and water of soil change, the catalytic ability of soil enzymes will change [33].

### 4.3. Effects of Atrazine on Carbon Source Utilization of Microbial Community in Cold Chernozem

The factors affecting the diversity of soil microbial carbon source utilization are mainly divided into natural factors and human interference [34]. In this study, Biolog technology was used to study physiological maps at the microbial community level in different cultivated soil layers of chernozem in a cold region. The results show that the functional diversity of the microbial community in three cultivated soil layers changed with time after atrazine application. This reflects that the activity of soil microorganisms in the natural state will be affected by time and space, resulting in corresponding changes in the average color change rate of carbon source (AWCD), functional diversity index, and diversity characteristics of carbon source utilization. This may be due to changes in soil temperature and the intensity of vegetation root activity and the natural succession law of natural rainfall with time. As for the spatial change, it may be related to the humification degree in different soil layers, the soil aeration conditions, and the weakened microbial utilization efficiency of litter components on the soil surface with the deepening of soil layers, which forms the succession law of soil microbial community functional diversity in time and space.

It was reported that the root activity of herbaceous vegetation and the existence of abundant litter on the surface expand the diversity of microorganisms in the topsoil [35]. In addition, soil fertilization and nutrient input can effectively improve the diversity of the soil microbial community. At the same time, the diversity characteristics of soil microorganisms in spring, summer, autumn, and winter are also different. In general, higher temperature and vigorous microbial metabolic activity in summer are conducive to the formation and accumulation of microbial diversity; low temperature in winter is conducive to the metabolic activity of low-temperature bacteria in soil [36]. This is consistent with the results of this study.

In this study, atrazine inhibited the diversity and overall activity of microorganisms in the 0–10 and 20–30 cm soil layers, but not in the 10–20 cm layer. This shows that the application of pesticides changes the inherent environmental conditions in the soil, and soil microorganisms must respond accordingly. At the initial stage of application, the effect of pesticides on microorganisms was significant, but over time, the effect gradually weakened, and the functional activity and diversity of soil microorganisms gradually recovered. In addition, due to the differences in the composition of the microbial community and the diversity of microbial carbon source utilization in different tillage soil layers, the stability of the microbial community in different soil layers and the resistance to pesticides are different, which eventually leads to differences in their metabolic activity affected by pesticides.

Regarding the effect of atrazine, the highest total activity of microorganisms at 1, 7, 21, and 119 d was in the 10–20, 0–10, 0–10, and 10–20 cm soil layers, respectively, and the lowest total activity was in the 20–30 cm layer. This result is consistent with the conclusion on the carbon source metabolism characteristics of microbial communities in three soil types and different depth soil profiles in Northeast China: the microbial activity in the 0–10 and 10–20 cm soil layers of farmland was very close, and the activity in the 0–20 cm layer was significantly higher [37]. This may be because the 0–20 cm soil layer is ploughed every year, and the roots of crops are mainly concentrated in this layer, while in the deeper layer below this, a hard plough layer is formed due to ploughing and mechanical compaction, which hinders the growth of plant roots and the transmission of water and fertilizer, resulting in the low activity of the soil microbial community below the plough layer. However, the distribution of microorganisms in 0–20 cm is relatively uniform, and the activity is relatively close, but generally higher than that in 20–30 cm [38].

In addition, from the metabolic results of microbial carbon sources, the relative utilization of the six carbon sources in the Biolog microplate by soil microorganisms in cultivated soil layers at different periods in the cold chernozem was not affected by atrazine, which may be because long-term continuous cropping leads to better adaptation of soil microorganisms in this environment. Soil microbial carbon metabolism functional groups have stronger carbon source metabolic activity, which forms stable characteristics and has strong resistance to changes from external environmental factors [39]. However, from the whole experiment, the utilization of the six carbon sources by microorganisms in different layers of atrazine-contaminated soil was in the order of sugars > carboxylic acids > amino acids > multi-clusters > amines > phenols. It can be seen that the utilization intensity of phenols and amines is relatively low, and the utilization of sugars, carboxylic acids, and amino acids is the highest. This is consistent with the conclusion on the characteristics of microbial community carbon source metabolism in three soil types and different depth soil profiles in Northeast China [37]. This may be due to the fact that the first several carbon sources can directly participate in the life activities of soil microorganisms and are more easily used by most microorganisms.

In addition, although the Biolog technology used in this study is simple and widely used in the study of soil microbial functional diversity, it has limitations. The carbon source added in the Biolog microplate does not include all kinds of compounds in the soil, which has a great artificial tendency. Moreover, only fast-growing microbial species can be detected, so the actual metabolic level of the soil microbial community may be underestimated [26]. Therefore, based on Biolog, this study also used high-throughput sequencing technology to further study the microbial community, functional diversity, and other aspects in order to provide more proof from the molecular, physiological, and biochemical levels and obtain more comprehensive and objective research results.

### 4.4. Effects of Atrazine on Bacterial Population and Abundance in Cultivated Soil Layers

High-throughput MiSeq sequencing technology was used to analyze the changes of microbial community composition and diversity in atrazine-contaminated soil. The results show that the composition of bacterial communities in the three soil layers was basically the same at each classification level. Atrazine did not change the bacterial community population but slightly changed the relative abundance of bacterial groups. The bacterial community composition of atrazine-contaminated soil mainly covered 12 phyla. According to the composition and distribution of bacterial communities in the three soil layers, *Proteobacteria*, *Actinobacteria*, *Actinomycetes*, and *Bumetomonas* were the dominant bacteria, accounting for 79.3% of the total relative abundance in the samples. This result is basically consistent with the conclusion that *Actinomycetes* and *Proteus* are the dominant phyla in the topsoil (0–20 cm) of long-term fertilized farmland in the Fukang area of Xinjiang (the relative abundance of the two phyla is about 48–65%) [40]. However, in the subsurface layer, the relative abundance of *Actinomycetes* gradually decreases and Proteus gradually increases (Figure S4A–C). This result is different from the variation of relative abundance of each phylum in each cultivated soil layer over time [41]. The research shows that there are great differences in microbial community composition between surface and subsurface soil (soils derived from Paleoproterozoic biotite schist and biotite gneiss bedrock) in the Gordon Canyon watershed profile (luvisols), Colorado, USA [42]. These studies show that the composition of the soil microbial community in different depths of the soil profile is different, and it can be inferred that the metabolic diversity of carbon sources by microorganisms in different depths of the soil profile may also be different.

In different soil layers, the effect of atrazine on the bacterial abundance of CK and AT groups at the genus level showed a change trend from insignificance to significance and then back to the CK level. In different soil layers, with the application of atrazine, the abundance of *Nitrospira*, *Pseudomonas*, *Sulfuricurvum*, *Acinetobacter*, *Flavobacterium*, *Salinibacterium*, and *Pedobacter* in AT groups increased significantly compared with CK groups. These bacteria play an important role in the degradation of pesticides [43], heavy metals [44], lignin [45], perfluorooctane sulfonic acid [46], polycyclic aromatic hydrocarbons [47], polychlorinated biphenyls [48], and cellulose [49], which may be the reason for the increase in abundance of these bacteria after atrazine application for a period of time.

According to the analysis of five alpha diversity indices of different soil layers (ACE, Chao1, observed number of species, PD whole tree, and Shannon), atrazine inhibited the diversity of bacterial communities in three cultivated soil layers, which was consistent with the results of Biolog data analysis. It should be noted that in the 10–20 cm soil layer, atrazine did not inhibit the overall activity of soil microorganisms, but inhibited their diversity, which may be related to the different microbial groups in the soil layer. Atrazine may have stimulated the growth of some dominant microbial groups, but it inhibited the activity of other nondominant microbiota in the samples. In the end, the overall microbial activity in the samples was not decreased, but the species diversity was reduced.

## 5. Conclusions

The results show that atrazine residues in tillage soil layers at different periods decreased with time (4.645–0.066 mg/kg soil). Atrazine promoted urease and polyphenol oxidase activity in the soil layers at different periods after application but had no effect on sucrase and phosphatase activity. Atrazine inhibited the diversity and overall activity of microorganisms in the 0–10 and 20–30 cm soil layers but only inhibited diversity in the 10–20 cm layer. Atrazine did not affect the relative utilization of carbohydrates, carboxylic acid, amino acids, multi-clusters, amines, and phenol carbon sources by soil microorganisms in the cultivated soil layers at different periods in the cold chernozem. Atrazine did not change the bacterial community population (phyla 10–12, genera 29–34) but slightly changed the relative abundance of each bacterial group. The inhibitory effect of atrazine on bacterial community diversity in cultivated soil layers of chernozem in the cold region decreased with the decrease in atrazine residue. In conclusion, the residual level of atrazine over time in a year has a certain influence on enzyme activity and microbial community population, function, and diversity in cultivated soil layers in a maize planting area in chernozem.

## Figures and Tables

**Table 1:** Correlation analysis of functional diversity of soil microbial community and atrazine residue and enzyme activity.

	A	B	C	D	E	F	G	H	J	K	L	M	N	O	P
A	1														
B	−0.023	1													
C	−0.242	0.261	1												
D	0.387	0.542 **	0.032	1											
E	0.216	0.613 **	0.13	0.728 **	1										
F	−0.161	−0.112	0.306	−0.439 *	−0.0242	1									
G	0.096	0.228	0.104	0.532 **	0.417	−0.620 **	1								
H	0.227	0.088	−0.456 *	0.174	0.043	−0.443 *	−0.084	1							
J	−0.093	−0.218	−0.268	−0.048	−0.212	−0.221	−0.1	−0.255	1						
K	−0.219	0.055	0.124	−0.165	0.049	−0.153	−0.328	0.295	−0.029	1					
L	0.209	0.009	0.069	−0.044	−0.007	0.065	0.041	−0.045	−0.583 **	−0.272	1				
M	0.410 *	0.32	0.074	0.638 **	0.271	−0.324	0.269	0.261	0.054	−0.281	0.016	1			
N	−0.486 *	−0.551 **	0.022	−0.723 **	−0.620 **	0.304	−0.582 **	0.053	0.103	0.342	−0.107	−0.394	1		
O	0.006	−0.363	−0.092	−0.38	−0.523 **	0.036	−0.488 *	0.32	0.115	0.187	0.056	0.099	0.682 **	1	
P	0.411*	−0.29	−0.235	−0.2	−0.337	−0.045	−0.432 *	0.515 *	−0.083	0.308	0.087	0.116	0.375	0.608 **	1

* Significant correlation at 0.05 level (bilateral), ** significant correlation at 0.01 level (bilateral). A: invertase; B: urease; C: phosphatase; D: polyphenol oxidase; E: atrazine residue; F: saccharides; G: carboxylic acids; H: amino acids; J: polymers; K: phenolics; L: amines; M: AWCD; N: Shannon; O: Simpson; P: McIntosh. Each value represents mean ± SD (n = 3).

## Data Availability

The data presented in this study are openly available on FigShare at doi:10.6084/m9.figshare.16443516.

## Supplementary Materials

The following are available online at https://www.mdpi.com/article/10.3390/microorganisms9091832/s1, Figure S1: Sampling diagram. Figure S2: Effect of atrazine on microbial community index of tillage soil layers in black soil. Figure S3: Effect of soil microbial community on relative utilization efficiency of carbon sources. Figure S4: Community population of soil samples at the phylum level. Figure S5: Heatmaps of the top 30 microbial phyla. Figure S6: Principal component analysis. Figure S7: Wayne maps. Table S1: Classification of carbon sources on Biolog microplate.
