# Supplementary material for: Effects of Atrazine on Chernozem Microbial Communities Evaluated by Traditional Detection and Modern Sequencing Technology
Source: Microorganisms. 2021 Aug 29;9(9):1832. doi: 10.3390/microorganisms9091832 (PMC8464665; doi:10.3390/microorganisms9091832)
Supplement: Supplementary file 1 [file microorganisms-09-01832-s001.zip › microorganisms-1336064-supplementary.pdf]

Supplementary material

1. Experimental design and soil samples

The specific sampling method of the test field was shown in Figure S1.

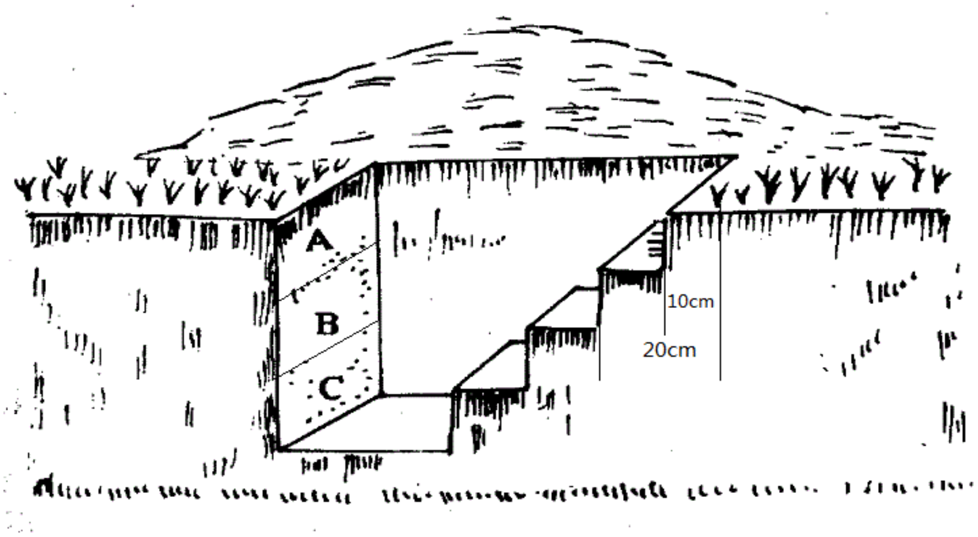

Figure S1. Sampling diagram. A: 0–10 cm; B: 10–20 cm; C: 20–30 cm.

2. Effect of atrazine on carbon utilization of microbial community in chernozem in cold region

According to the method in Section 2.3, the absorbance values of soil samples cultured for 72 h were selected to calculate Shannon, Simpson, and McIntosh indices to evaluate the changes in microbial community richness, the dominance of the most common microbial, and the microbial homogeneity with atrazine in cultivated soil layers at different times in a cold region.

In the 0–10 cm soil layer, compared with CK groups, the Shannon index of AT groups decreased at 7 d and returned to the CK group level at 21 and 119 d. The Simpson index of the AT groups had no significant change at 1, 7, and 21 d, but increased significantly at 119 d. The McIntosh index of the AT groups decreased significantly at 1 d and returned to the CK group level at 7, 21, and 119 d ($p < 0.05$).

In the 10–20 cm soil layer, compared with the CK groups, the Shannon index of the AT groups had no significant change on the first day, or at 7, 21, and 119 d. The Simpson index of the AT groups decreased significantly at 21 d and returned to the CK level at 119 d. The McIntosh index of the AT groups decreased significantly on the first day, returned to the CK group level at 7 and 21 d, and increased significantly at 119 d ($p < 0.05$).

In the 20–30 cm soil layer, compared with the CK groups, the Shannon index of the AT groups had no significant change at 1, 7, 21, and 119 d. The Simpson index of the AT groups increased significantly on the first day, returned to the level of the control group at 7 d, decreased significantly at 21 d, and increased significantly at 119 d. The McIntosh index of the AT groups decreased significantly at 21 d and returned to the CK group level at 119 d ($p < 0.05$).

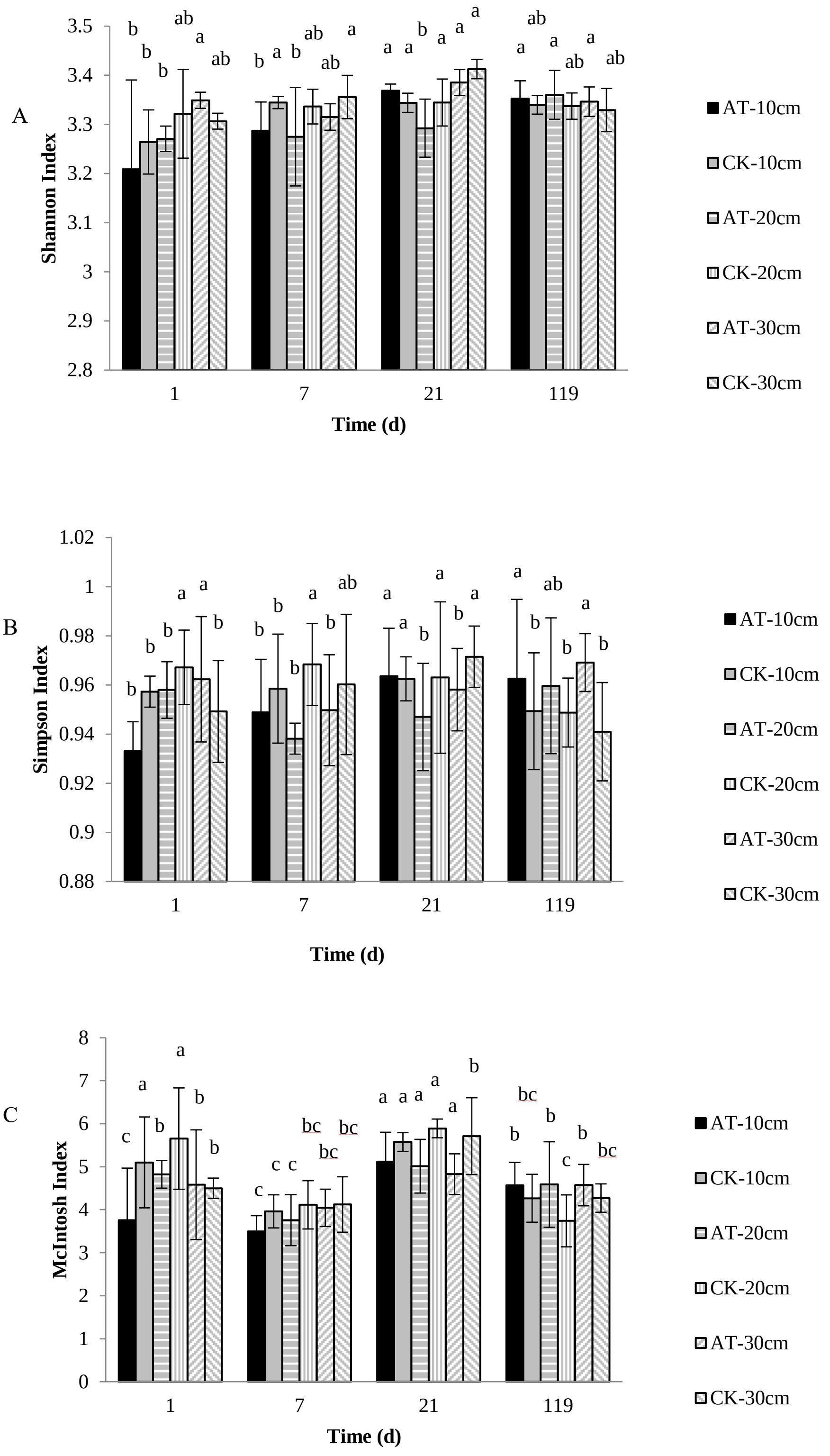

Figure S2. Effect of atrazine on microbial community index of tillage soil layers in black soil. (A) Shannon index, (B) Simpson index, (C) McIntosh index. Each value represents mean ± SD (n = 3). There was no significant difference between CK and AT groups ($p > 0.05$), but there was a significant difference between CK groups and AT groups ($p < 0.05$).

The results show that compared with CK groups, AT groups had little effect on the relative utilization rate of carbon sources at 1, 7, 21, and 119 d, and in the whole experiment, the utilization degree of the six carbon sources by microorganisms was in the order of sugars > carboxylic acids > amino acids > multi-clusters > amines > phenols. Atrazine had little effect on the carbon metabolism of soil microorganisms.

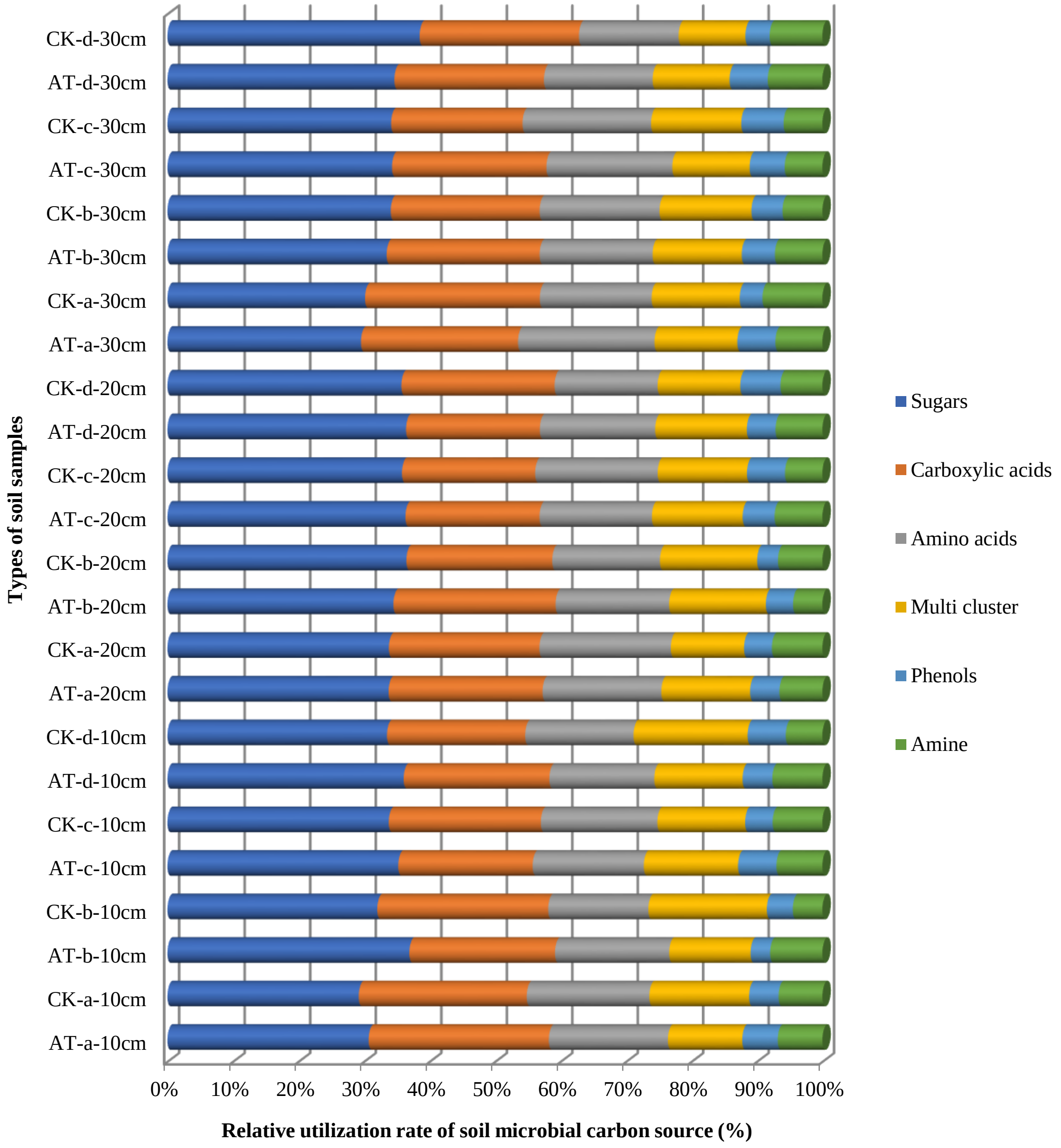

Figure S3. Effect of soil microbial community on relative utilization efficiency of carbon sources. (a) 1 d, (b) 7 d, (c) 21 d, (d) 119 d. Each value represents mean ± SD (n = 3).

3. Effect of atrazine on bacterial population in cultivated soil layers

The phyla composition analysis results of microbial community at the classification level of atrazine in different periods and different cultivated soil layers of cold black soil show that there are mainly 12 phyla of bacterial community in 0-10cm soil layer, which are *Proteobacteria* and *Acidobacteria* from high to low, *Actinobacteria*, *Gemmatimonadetes*, *Bacteroides*, *Chloroflexi*, *Verrucomicrobia*, *Nitrospirae*, *Phytomycetes*, *Firmicutes*, TM7, *Cyanobacteria*. Compared with 0-10cm soil layer, the phyla composition of 10-20cm and 20-30cm soil layer lacks TM7 and *Cyanobacteria*. From the composition and distribution of bacterial communities in the three soil

layers, *Proteus*, *Acidobacteria*, *Actinomycetes* and *Blastomonas* are the dominant bacteria, accounting for about 79.56% of the total relative abundance of the sample.

The composition of bacterial communities in the three soil layers is basically the same at the phylum level, but the relative abundance of groups is slightly different. With the passage of application time, atrazine changed the relative abundance of bacterial community in different periods and cultivated soil layers of cold black soil. In the 0-10cm soil layer, the relative abundance of *Acidobacteria* and *Gemmatimonadetes* is opposite to the residual change law of atrazine in the same soil layer in different periods after application. With the passage of application time, the relative abundance gradually increases, with the increase rates of 96.8% and 209.3% respectively, while cyanobacteria, proteus. The change trend of relative abundance of the three phyla of *Cyanobacteria*, TM7 and *Proteobacteria* consistent with that of atrazine residue in the same soil layer in different periods. With the passage of application time, the relative abundance gradually decreased, and the decline rates were 94.6%, 45.4% and 60.85% respectively. The relative abundance of *Bacteroidetes* remained unchanged with the passage of application time. In 10-20cm soil layer, the change trend of *Proteus* is consistent with that of atrazine residue in the same soil layer in different periods of cold black soil, and the change trend of *Gemmatimonadetes* is completely opposite to that of atrazine residue in the same soil layer in different periods of cold black soil. The relative abundance of *Acidobacteria* increased continuously with the application time, and the growth rate was 121.6%. The relative abundance of *Firmicutes* decreased continuously with the application time, and the decline rate was 62.2%. *Proteus*, *Acidobacteria*, *Actinobacteria* and *Gemmatimonadetes* are the dominant bacteria, accounting for about 80.5% of the total relative abundance of the sample. In 20-30cm soil layer, the change trend of *Firmicutes* is consistent with that of atrazine residue in the same soil layer in different periods of cold black soil, and the change trend of *Actinobacteria* is completely opposite to that of atrazine residue in the same soil layer in different periods of cold black soil. The relative abundance of *Acidobacteria* and *Gemmatimonadetes* increased continuously with the application time, and the growth rates were 87.97% and 215% respectively. The relative abundance of *Chloroflexi* decreased continuously with the passage of application time, and the decline rate was 67.5%. *Proteus*, *Acidobacteria*, *Actinobacteria* and *Gemmatimonadetes* are the dominant bacteria, accounting for about 77.8% of the total relative abundance of the sample (Figure S4).

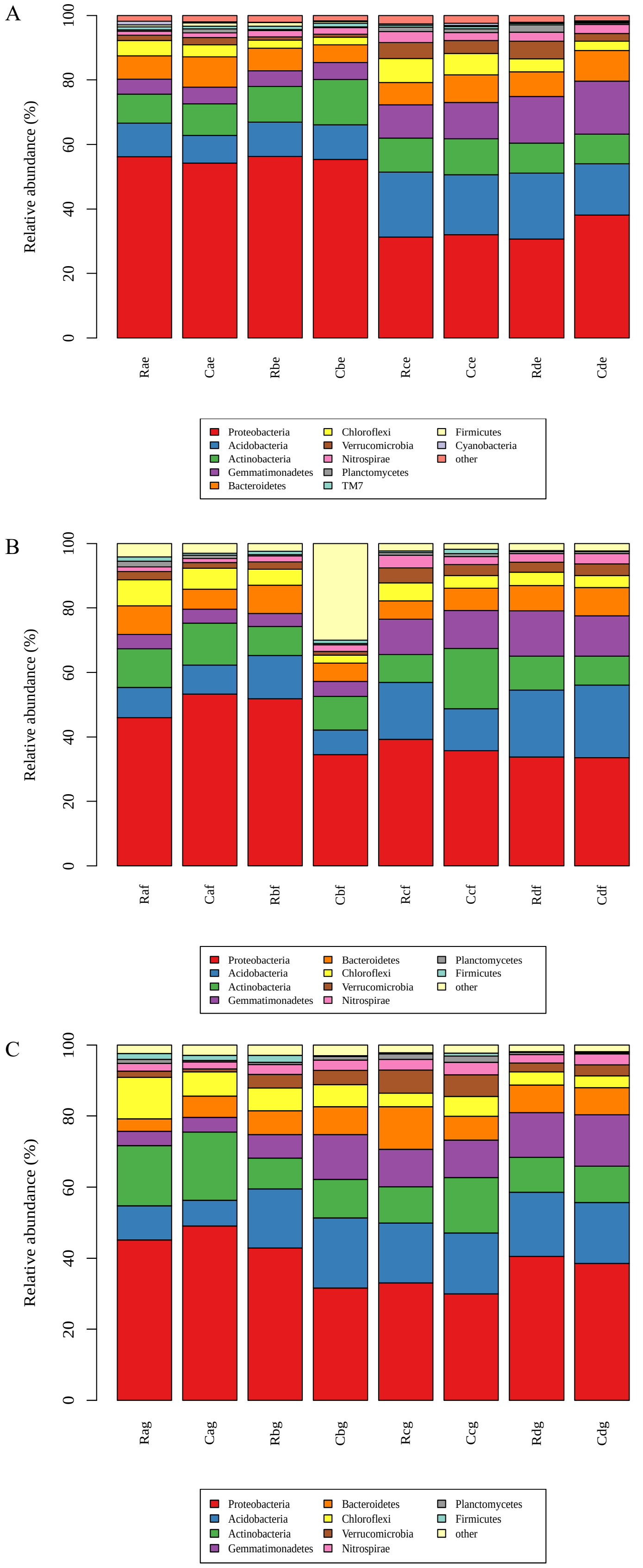

Figure S4. Community population of soil samples at phylum level. (A) 0–10 cm, (B) 10–20 cm, (C) 20–30 cm. Each value represents mean ± SD (n = 3). R, C indicate atrazine in AT and CK at recommended dose, respectively; a, b, c, d indicate soil samples at 1, 7, 21, and 119 d, respectively; e, f, g indicate 0–10, 10–20, and 20–30 cm soil layers, respectively.

Bacterial heatmap analysis was carried out according to the phyla composition and relative abundance of samples in different periods and cultivated soil layers of chernozem in a cold region, and the 30 phyla with the highest abundance were extracted at the taxonomic level for heatmap cluster analysis.

In the 0–10 cm soil layer, AT groups (Rae, Rbe) at 1 and 7 d of application were classified into two phyla, indicating that atrazine had a certain effect on the population composition and relative abundance of bacteria in this layer. However, with decreased atrazine residue, this effect gradually weakened, and AT groups (Rce, Rde) at 21 and 119 d were classified into one phylum. This shows that the similarity is very high, but there are still some differences between them and the CK groups.

In the 10–20 cm soil layer, AT groups (Raf, Rbf) at 1 and 7 d of application were classified into two phyla, indicating that atrazine residues gradually accumulated with increased soil depth and reached the maximum value after 7 d, which had different effects on the composition of the bacterial community and the relative abundance of phyla in the soil layer in different periods. The greater the atrazine residue, the greater the impact on the bacterial community. However, with decreased atrazine residue, this effect gradually weakened. AT groups (Rcf, Rde) were classified into the same group at 21 and 119 d of atrazine application, indicating that the similarity was very high. At 119 d, AT groups (Rdf) and CK groups (Cdf) were classified into one group, indicating that atrazine had little effect on the soil bacterial community at the later stage of application.

The effect of atrazine on the bacterial community composition and relative abundance of phyla in the 20–30 cm soil layer in different periods was basically the same as in the 10–20 cm layer. The AT groups (Rag and Rbg) at 1 and 7 d of application were classified into two phyla, indicating that atrazine residues gradually accumulated with increased soil depth and reached the maximum value after 7 d. The greater the atrazine residue, the greater the impact on the bacterial community. However, with decreased atrazine residue, this effect gradually weakened. AT groups (Rcg, Rdg) at 21 and 119 d were classified into the same group, indicating that the similarity was very high and the effect of atrazine was very small (Figure S5).

Principal component analysis (PCA) between samples showed that in the 0–10, 10–20, and 20–30 cm tillage soil layers, atrazine application had similar scores on the PC1 axis at 1 and 7 d compared with control. In the PCA diagram, soil with applied atrazine and control soil basically gather in a cluster, and the distance is very close. The composition of samples was similar, indicating that atrazine did not change the soil bacterial community. At the later stage of application, with the passage of time and the digestion or leaching of atrazine residues, the soil bacterial community had a natural evolution, resulting in dispersion of the community (Figure S6).

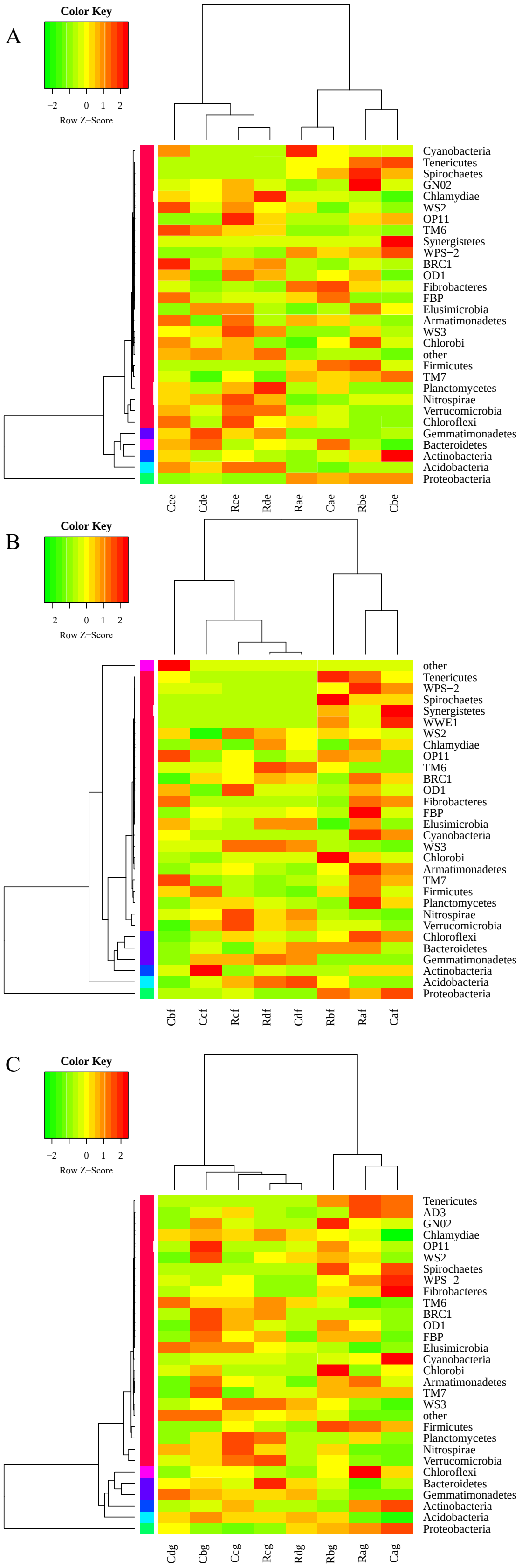

Figure S5. Heatmaps of top 30 microbial phyla. (A) 0–10 cm, (B) 10–20 cm, (C) 20–30 cm. Each value represents mean ± SD (n = 3). R, C indicate atrazine in AT and CK at recommended dose, respectively; a, b, c, d indicate soil samples at 1, 7, 21, and 119 d, respectively; e, f, g indicate 0–10, 10–20, and 20–30 cm soil layers, respectively.

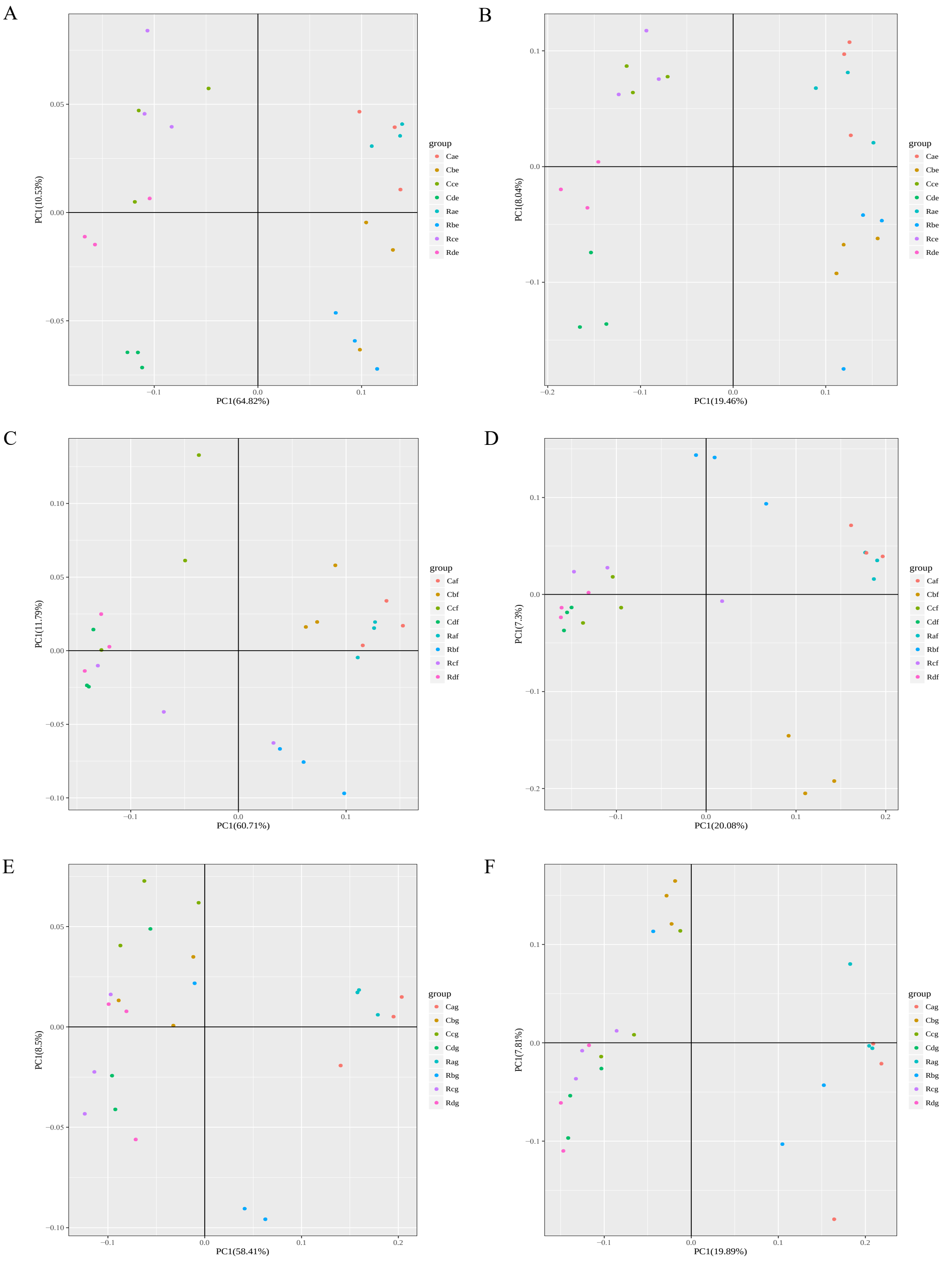

Figure S6. Principal component analysis. weighted_unifrac and unweighted_unifrac, respectively, of (A,B) 0–10 cm; (C,D) 10–20 cm; (E,F) 20–30 cm. R, C indicate atrazine treatment group and control group with recommended dose, respectively. Each value represents mean ± SD (n = 3). a, b, c, d indicate soil samples at 1, 7, 21, and 119 d after spraying, respectively.

There were 1977 OTUs in atrazine AT groups in the 0–10 cm soil layer at 1, 7, 21, and 119 d, which was 23 OTUs more than CK groups. The specific OTU number among AT groups showed a trend of increasing at the early stage and decreasing later, which was completely opposite to the trend of CK groups, and also contrary to the residual change trend of atrazine in the same soil layer at the same time. This indicates that atrazine could increase the total OTU number in soil and inhibit the specific OTU number among the groups, which may be due to the different response or sensitivity of different microorganisms in the soil to atrazine (Figure S7 A, B). It can be seen that there were 1770 OTUs in the atrazine AT groups in the 10–20 cm soil layer at 1, 7, 21, and 119 d, which was 175 OTUs more than CK groups. The number of OTUs in AT groups showed a trend of increasing in the early stage, but opposite to the CK groups, and also contrary to the residual trend of atrazine in the same soil layer at the same time. This indicates that atrazine could increase the total OTU number in soil and inhibit the specific OTU number among the groups (Figure S7 C, D). It can be seen that there were 1982 OTUs in the atrazine AT groups in the 20–30 cm soil layer at 1, 7, 21, and 119 d, and 1991 OTUs in the CK groups. The lower number of OTUs in AT than CK groups indicates that atrazine inhibited the number of OTUs (Figure S7 E, F). In a word, atrazine has a certain inhibitory effect on OTUs in cultivated layers of chernozem in a cold region and, to some extent, can increase the number of OTUs.

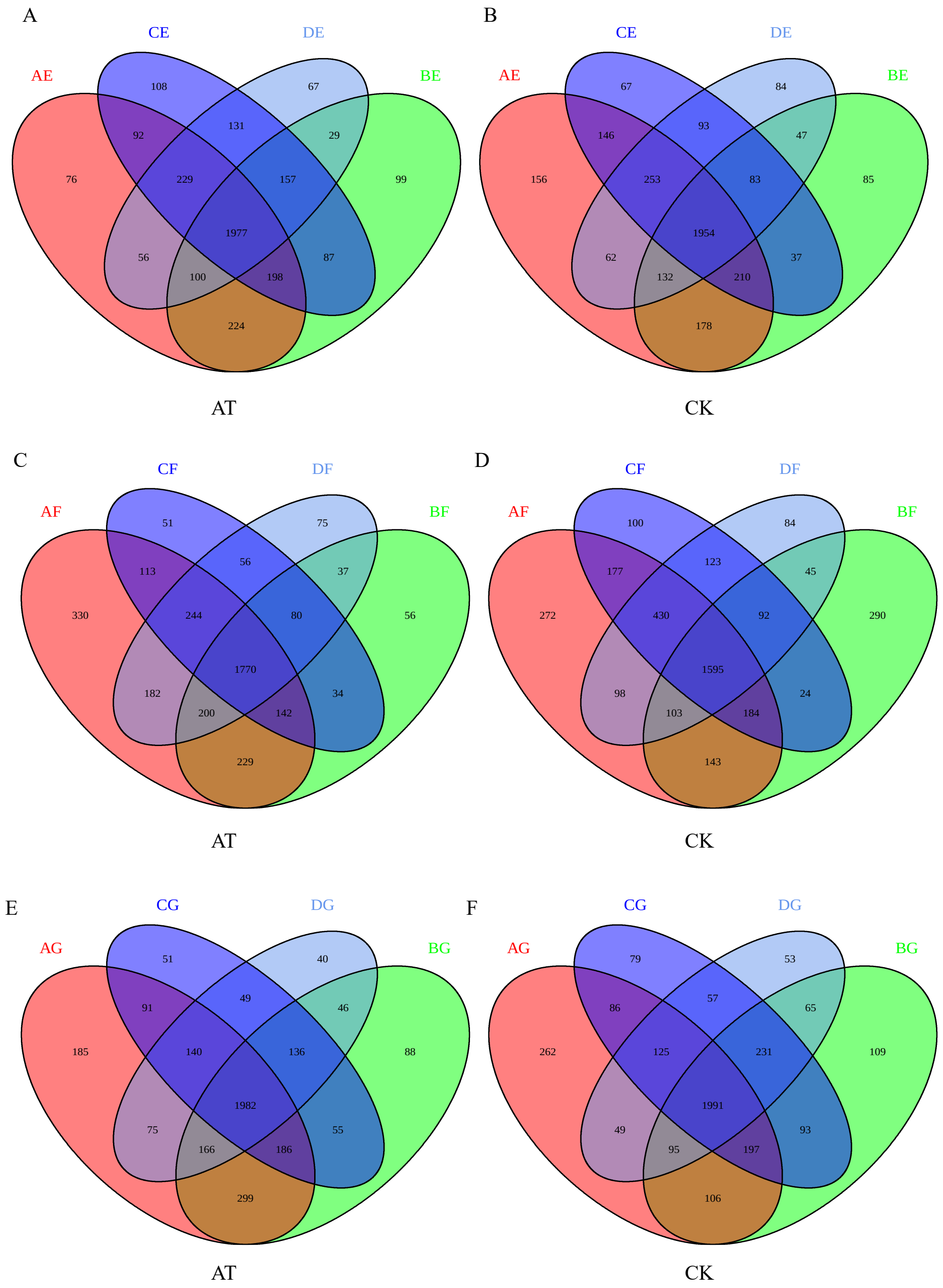

Figure S7. Wayne maps of (A,B) 0–10 cm, (C,D) 10–20 cm, and (E,F) 20–30 cm soil layers. AT: treatment group with atrazine; CK: control group without atrazine; AE/BE/CE/DE, AF/BF/CF/DF, and AG/BG/CG/DG: samples of 0–10, 10–20, and 20–30 cm soil layers taken at 1, 7, 21, and 119 d after application, respectively. Each value represents mean ± SD (n = 3).

4. The effect of atrazine on the functional diversity of soil microbial community measured by Biolog

Table S1. Classification of carbon sources on Biolog microplate.

Sugars	Amino acids	Carboxylic acids	Multi cluster	Phenols	Amine
β-methyl-D-glucoside	L-arginine	γ-Hydroxybutyric acid	α-cyclodextrin	2-Hydroxy benzoic acid	Phenylethylamine
D–xylose	L-asparagine	Itaconic acid	Glycogen	4-Hydroxy benzoic acid	Putrescine
i-Erythritol	L-phenylalanine	α-butyric acid	Tween 40		
D-mannitol	L-serine	D-malic acid	Tween 80		
N-acetyl-D-glucosamine	L-threonine	Pyruvic acid Methyl ester			
α-D-glucose-l-phosphate	Glycyl-L-glutamic	D-glucosaminic acid			
D, L-α-glycerolphosphate		D-galactonic acid			
D-cellobiose					
α-D-lactose					
D-galactoside					
